# Multiple Chaperone DnaK–FliC Flagellin Interactions are Required for *Pseudomonas aeruginosa* Flagellum Assembly and Indicate a New Function for DnaK

**DOI:** 10.1111/1751-7915.70096

**Published:** 2025-02-12

**Authors:** Gabriella Molinari, Sara S. Ribeiro, Katrin Müller, Benjamin E. Mayer, Manfred Rohde, Alejandro Arce‐Rodriguez, Juan José Vargas‐Guerrero, Albert Avetisyan, Josef Wissing, Werner Tegge, Lothar Jänsch, Mark Brönstrup, Antoine Danchin, Martina Jahn, Kenneth N. Timmis, Simon Ebbinghaus, Dieter Jahn, José Manuel Borrero‐de Acuña

**Affiliations:** ^1^ Central Facility for Microscopy Helmholtz Centre for Infection Research (HZI) Braunschweig Germany; ^2^ Institute of Physical and Theoretical Chemistry Technische Universität Braunschweig Braunschweig Germany; ^3^ Institute of Microbiology Technische Universität Braunschweig Braunschweig Germany; ^4^ Computational Biology and Simulation Technische Universität Darmstadt Darmstadt Germany; ^5^ Department Cellular Proteome Research Helmholtz Centre for Infection Research (HZI) Braunschweig Germany; ^6^ Department of Chemical Biology Helmholtz Centre for Infection Research (HZI) Braunschweig Germany; ^7^ School of Biomedical Sciences, Li KaShing Faculty of Medicine The University of Hong Kong SAR Hong Kong China; ^8^ Integrated Centre of Systems Biology (BRICS) Technische Universität Braunschweig Braunschweig Germany

**Keywords:** DnaK, flagellum, FliC, Hsp70, *Pseudomonas aeruginosa*

## Abstract

The DnaK (Hsp70) protein is an essential ATP‐dependent chaperone foldase and holdase found in most organisms. In this study, combining multiple experimental approaches we determined FliC as major interaction partner of DnaK in the opportunistic bacterial pathogen *Pseudomonas aeruginosa* . Implementing immunofluorescence microscopy and electron microscopy techniques DnaK was found extracellularly associated to the assembled filament in a regular pattern. *dnaK* repression led to intracellular FliC accumulation and motility impairment, highlighting DnaK essentiality for FliC export and flagellum assembly. SPOT–membrane peptide arrays coupled with artificial intelligence analyses suggested a highly dynamic DnaK–FliC interaction landscape involving multiple domains and transient complexes formation. Remarkably, in vitro fast relaxation imaging (FReI) experiments mimicking ATP‐deprived extracellular environment conditions exhibited DnaK ATP‐independent holdase activity, regardless of its co‐chaperone DnaJ and its nucleotide exchange factor GrpE. We present a model for the DnaK‐FliC interactions involving dynamic states throughout the flagellum assembly stages. These results expand the classical view of DnaK chaperone functioning and introduce a new participant in the *Pseudomonas* flagellar system, an important trait for bacterial colonisation and virulence.

## Graphical abstract

The dogma on the heat shock DnaK chaperone establishes its fundamental cytosolic role in folding and unfolding intracellular proteins. This study reveals its tight association with the flagellin, its secretion, likely through the flagellum apparatus, its insertion into a growing flagellum filament and a novel extracellular ATP‐independent chaperone‐folding functioning (Created with BioRender.com).

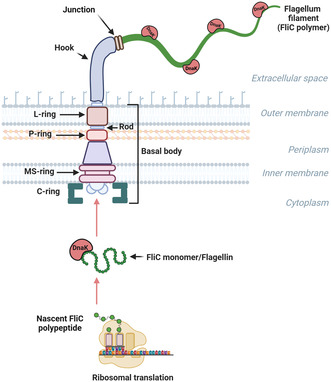

## 1 Introduction

The heat shock protein 70 (Hsp70) class of chaperones are proteins essential for a wide range of cellular housekeeping functions in all domains of life. They guide the folding of newly synthesised proteins and accompany the translocation of polypeptides into chloroplasts, mitochondria and the endoplasmic reticulum. One major function of all Hsp70 proteins is counteracting accumulation of aggregates of misfolded denatured proteins by binding to and solubilising the aggregates. Such substrates bound by the chaperones are either refolded to their native states or targeted for degradation by the appropriate protease system (Nillegoda, Wentink, and Bukau 2018) preserving cellular protein homeostasis by preventing proteotoxic stresses (Fernández‐Fernández and Valpuesta 2018).

**FIGURE 1:**
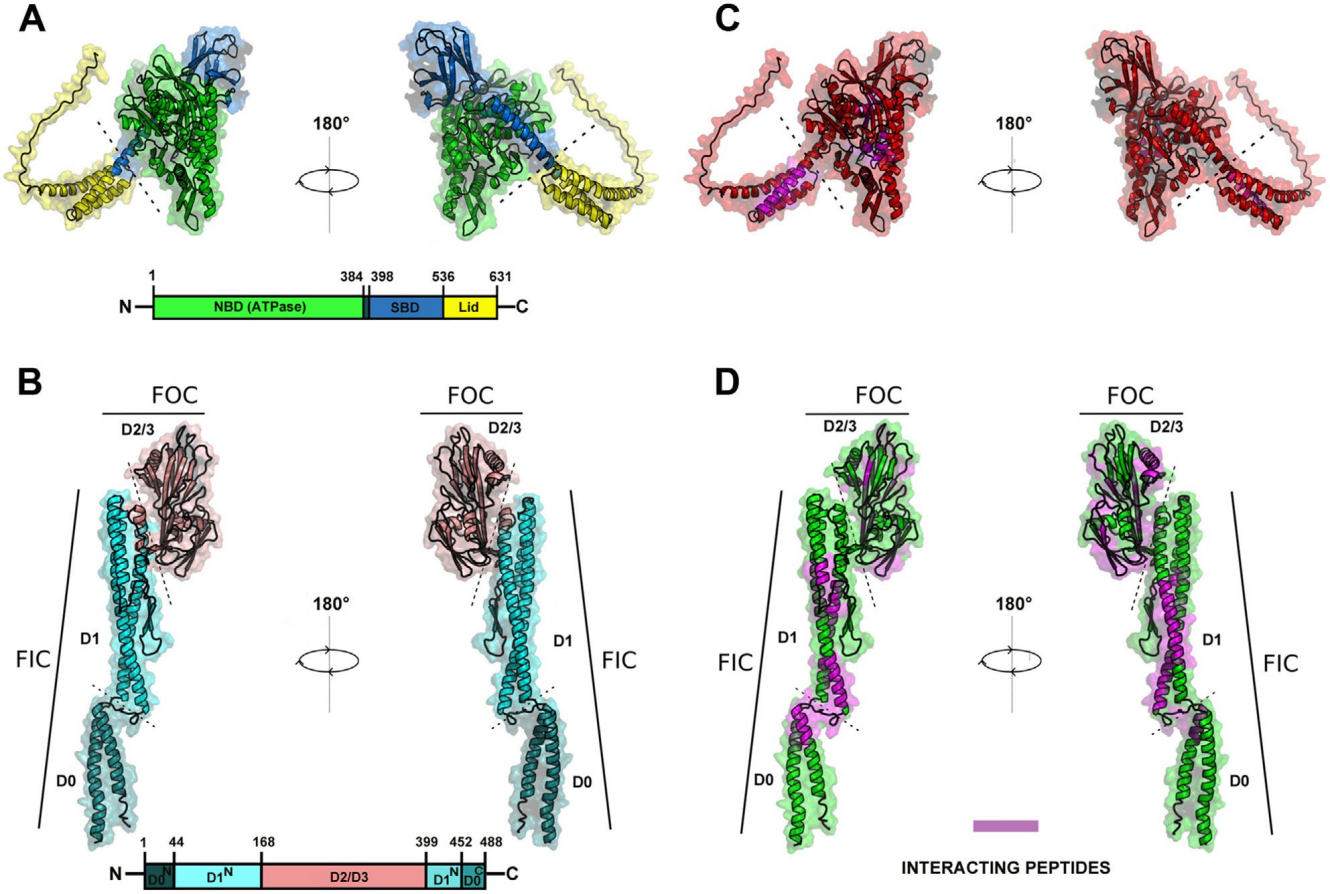
3D structures of DnaK and FliC and their interacting peptides. The protein structure of both proteins of interest are illustrated here (A–D). DnaK is shown in (A) where the nucleotide binding domain (NBD, Residues 1–384) is coloured in green, followed by the substrate‐binding domain (SBD, Residues 398–536) in blue and finally the C‐terminal lid (Residues 537–637) in yellow. The structure of FliC is illustrated in (B) where the D0 domain (Residues 1–44 and 452–488) is coloured in dark green and the D1 domain (Residues 44–168 and 399–452) is coloured in cyan, which form the tubular lower part of FliC. The previously unresolved D2/D3 region (Residues 168–399) is coloured in light red. FOC, filament outer core; FIC, filament inner core. In (C) and (D) the interacting peptides found in this study (Table 2) are coloured magenta in both structures.

The bacterial counterpart to eukaryotic Hsp70 is designated DnaK (Bertelsen et al. 2009). The highly conserved protein structure consists of a ca. 40 kDa N‐terminal nucleotide‐binding domain (NBD) connected via a linker to a ca. 25 kDa C‐terminal substrate binding domain (SBD) (Figure 1). The SBD is formed by β‐sandwich domain (15 kDa) and C‐terminal α‐helical domain. The NBD, also named ATPase domain, is composed of two subdomains called I and II, both having regions a and b. A classical nucleotide binding fold is formed by Subdomains Ia and IIa (Genest, Wickner, and Doyle 2019; Rosenzweig et al. 2019). The DnaK reaction cycle starts with the binding of a denatured protein substrate by the dimeric DnaJ (Hsp40) chaperone subunit of the DnaJ–DnaK complex while ATP is loaded on the complex but not immediately hydrolysed forming a transient ternary energy‐loaded complex (Noguchi et al. 2014; Rosenzweig et al. 2019). The DnaK–ATP form is characterised by an open lid and a low affinity to the substrate. DnaJ–DnaK–ATP‐misfolded substrate interaction triggers ATP hydrolysis leading to the formation of the high affinity, closed lid DnaK–ADP–substrate complex with the release of DnaJ and *P*_*i*_, which stabilises an extended conformation of the substrate to allow for the subsequent correct refolding. In order to release the substrate from DnaK, bound ADP is released by the nucleotide exchange factor (NEF) GrpE, thereby resetting DnaK with its low substrate affinity and an open lid of the substrate binding site. The cycle is concluded with the release of the substrate and the regeneration of DnaK prone to interact with other substrates while loaded with ATP (Clerico et al. 2019; Mayer and Gierasch 2019). This opens up also a window of ATP‐independent activities where DnaK can interact with other substrates than DnaJ as witnessed by the evolution by gene duplication of the *dnaK* gene when the proteome diversity increases (Pan et al. 2024). Several other extracellular functions have been reported for DnaK in eukaryotes and prokaryotes. Hsp70 was discovered in the extracellular space and on the plasma membrane, where it stimulates the immune system (chaperokine) (Tukaj 2020), associates with tumour cells in the serum (Boudesco et al. 2018) and interacts with lipids. However, the molecular mechanism for the transport of Hsp70 through the cytoplasmic membrane is discussed controversially as an obvious leader peptide is missing. Hypotheses to account for this include co‐transport with other proteins, phosphatidylserine flipping, release after cell death and exosome export (Boudesco et al. 2018). In bacteria, studies of surface proteomes identified DnaK on the cell surface of various species interacting with plasminogen (Candela et al. 2010) and binding invertase (Katakura et al. 2010). These ‘moonlighting functions’ require DnaK to be exported from the cytoplasm to the cell surface (Wang and Jeffery 2016; Chen et al. 2021). In our previous study, we discovered DnaK in the periplasm of *Pseudomonas aeruginosa* as part of a triple protein complex with the flagellar filament structural protein FliC and the periplasmic nitrite reductase NirS (Borrero‐de Acuña et al. 2015). Under anaerobic, nitrate‐respiring conditions, flagellar assembly and swimming motility were found to be dependent on the NirS‐DnaK‐FliC complex in the periplasm. The unexpected complex formation between DnaK and FliC directed our work to further investigate the detailed molecular basis for the interplay of the two proteins and its consequences for motility.

The major motor protein complex of motility in bacteria is the freely rotating flagellum and its structure and assembly has been extensively studied in *Salmonella enterica* (Al‐Otaibi and Bergeron 2022; Minamino and Kinoshita 2023). In this organism, the flagellum is composed of multiple proteins forming ring structures (rings C, MS, P, L) that span the cytoplasmic membrane, the cell wall, the periplasm and the outer membrane. A basal body acts as a bidirectional rotary motor and the hook along with its junction proteins, serves as the universal joint for transmission of the torque produced by the force generators to the helical propeller of the filament (composed of FliC), which effects propulsion (Nedeljković, Sastre, and Sundberg 2021). The filament is capped by FliD, which promotes efficient and robust assembly of a newly transported FliC subunit. Ion flow‐reliant flagellar rotation is enabled by the assembly of several stator units around the basal body rotor ring complex. Assembly of the whole apparatus is mediated by an integrated ATP‐driven type III protein secretion system that transports structural proproteins from the cytoplasm to the appropriate position in the machinery (Halte and Erhardt 2021; Minamino, Kinoshita, and Namba 2022). The export channel inside of the flagellum is approximately two nm in diameter and thus can only allow for transport of partially unfolded proteins. The process of flagellar assembly is divided into several stages where the ordered transport of the various proteins through the growing apparatus is controlled by multiple chaperones. In peritrichous flagellated species, the dedicated chaperone FliS binds to FliC in the cytoplasm and transfers FliC to the Type III secretion system of the flagellum. FliS binds to the C‐terminal disordered region of FliC (Yokoseki et al. 1995; Ozin et al. 2003; Muskotál et al. 2010). In the absence of FliS, a short filament phenotype was observed.

The overall structure of the single polar flagellum of *P. aeruginosa* resembles that of the well‐investigated counterparts of peritrichous flagellated species (Bouteiller et al. 2021). However, regulation of the corresponding 60 genes differs significantly in *P. aeruginosa* . The present work shows that, in *P. aeruginosa* cells, DnaK is tightly bound to the filament protein FliC under multiple physiological conditions. It was also present in large quantities in the external environment, where it was found associated with the flagellar filament composed of FliC. These findings prompted a series of questions concerning the exact molecular nature of DnaK–FliC interaction, the presence of the DnaK protein outside of the cell, and its extracellular function in the absence of ATP. A novel ATP‐independent function of DnaK tested under acidic conditions in vitro was ascertained. The findings unveil new avenues for the investigation of potential DnaK functions beyond those chaperone roles confined to the cytoplasmic milieu.

## 2 Experimental Procedures

### 2.1 Genetic Manipulations and General Growth Conditions

All bacterial strains, primers and construct included in this study are listed in Table S1. For the construction of IPTG‐inducible plasmids harbouring the recombinant versions *dnaK*‐GST and *fliC*‐GST, the primers pairs of *dnaK*Fw/Rv and *fliC*Fw/Rv were used to amplify each gene, respectively. Subsequently, restriction sites were introduced using the previous amplicons as DNA templates and the primers *dnaK*FwBamHI/*dnaK*FwXhoI and *fliC*Fw_BamHI/ *fliC*Fw_XhoI in PCR reactions, respectively. Finally, the resulting amplicons were digested with BamHI and XhoI and ligated into the pGEX‐6P‐1, giving rise to pGEX‐6P‐1‐*dnaK* and pGEX‐6P‐1‐*fliC*, respectively. The *E. coli* BL21(*λ*DE3) strain served as a proliferation vehicle for the pGEX‐6P plasmid and subsequent protein production. *P. aeruginosa* UCBPP‐PA14 (Rahme et al. 1995) was the strain used in this study. Bacteria were grown aerobically in Lysogeny Broth (LB) medium at 37°C and 180 rpm, unless stated otherwise. *E. coli* cultures were supplemented with Ampicillin (100 μg/mL) for plasmid selection. For *P. aeruginosa* strains bearing transposon insertions gentamycin (50 μg/mL) was used only for overnight cultures, whereas no antibiotic was added for short term exponential cultivations.

### 2.2 Construction of a *dnaK* Conditional Mutant

To construct a *dnaK* conditional knock out mutant a *P. aeruginosa* strain bearing an inducible *dnaK* antisense RNA expression plasmid was constructed. For that purpose, an amplicon spanning the *dnaK* promoter region, its associated Shine–Dalgarno sequence and the overall stretch of the gene were amplified using the primers anti‐*dnaK*Fw_BamHI *and* anti‐*dnaK*Rv_EcoRI. Due to the selection of restriction enzymes included in the overhangs, the ligation of the resulting amplicon into EcoRI‐BamHI digested pSEVA634 resulted in pSEVA634‐antisense‐*dnaK* harbouring the above‐mentioned DNA sequence but with an inverted orientation resulting in complementary to the mRNA resulting from the transcription of the genomic *dnaK* gene sequence. This plasmid was introduced in *P. aeruginosa* PA14 resulting in the PA14KD*dnaK* strain. For induction of the synthesis of the anti‐*dnaK* RNA interference molecules, cells were grown as described above with 0.1 mM of IPTG and 1 mL samples were withdrawn over time at 0, 2, 4, 6, 8 and 24 h. Cells were pelleted at 13,000 rpm for 5 min, supernatants were discarded and the pellet was resuspended in 100 μL SDS‐loading buffer (15% [vol/vol] glycerol, 5% [vol/vol] β‐mercaptoethanol, 2.4% [wt/vol] SDS, 1% [wt/vol] bromophenol blue, 0.8% [wt/vol] Tris, pH 6.8) for further SDS‐PAGE analyses. Resolved proteins were transferred onto a polyvinylidene difluoride (PVDF) membrane. DnaK was chromogenically detected using anti‐DnaK primary polyclonal rabbit antibody at a 1:1000 dilution, whereas rabbit alkaline phosphatase conjugated secondary antibody (Sigma‐Aldrich, Taufkirchen, Germany) was added at a 1:10,000 dilution. The chromogenic reaction was developed using the alkaline phosphatase BCIP/NBT substrate as suggested by the manufacturer (Sigma‐Aldrich, Taufkirchen, Germany).

### 2.3 DnaK and FliC Production

A preculture with 6 mL LB medium (Ampicillin, 100 μg/mL) was inoculated with BL21 cells carrying plasmids pGEX‐6P‐*dnaK* or pGEX‐6P‐*fliC* and incubated at 37°C with 200 rpm agitation overnight. The main culture from 500 mL LB medium (Ampicillin, 100 μg/mL) was inoculated with 5 mL of the preculture and incubated at 37°C and 200 rpm agitation up to an OD_578_ of 0.4–0.6. Then, 200 μM of IPTG were added for induction of gene expression and the cultures were further incubated at 17°C with 200 rpm agitation overnight. Cells were harvested using centrifugation at 3500 rpm (Avanti J‐26, Beckham Coulter) for 20 min at 4°C. The cell pellet was resuspended with 10 mL 1 × PBS (137 mM NaCl, 2.7 mM KCl, 4.3 mM Na_2_HPO_4_∙7 H_2_O and 1.4 mM KH_2_PO_4_, pH 7.3) and the cell disruption was performed using a FrenchPress cell at 1200 psi. The lysate was centrifuged at 100,000 *g* at 4°C for 1 h and the supernatant (S100) free of cellular debris was collected. Five mL of Glutathione Sepharose 4B (Sigma‐Aldrich, Taufkirchen, Germany) were equilibrated with PBS. The sample was added to the column, gently mixed, and incubated for 2 h at 17°C for fusion protein binding. The material filled in a chromatographic column was washed four times with three column volumes of washing buffer. For the elution of the target protein, 500 units of prescission protease (Sigma‐Aldrich, Taufkirchen, Germany) were mixed with 7 mL PBS, added to the column material and mixed carefully. The column was re‐filled and stored at 17°C overnight for incubation. The protein was eluted first with 7 mL PBS and afterwards twice with 15 mL, collecting the resulting fractions. For the elution of the bound GST tag, 2.5 mM glutathione were solved in PBS, the column was washed twice, and fractions were collected.

### 2.4 Antibodies

The polyclonal antibodies were obtained previously (Borrero‐de Acuña et al. 2015) against the overproduced DnaK protein and synthetic peptides from the FliC protein. The respective IgGs were purified using a protein A sepharose column.

### 2.5 SPOT‐Membrane Array

The SPOT method for screening protein–protein interactions using peptide libraries on cellulose paper has been described previously in detail (Beutling et al. 2008). In brief, the DnaK‐GST and FliC‐GST recombinant proteins were purified as described above. The affinity tag was cleaved off by precision protease treatment prior to the addition of each protein to the membrane array. In this array, each spot on the membrane contains a synthetic peptide of 15 amino acids with an overlap of 12 amino acids to the next one (shifting the sequence by three amino acids), spanning the entire DnaK and FliC sequences from the N‐ to the C‐terminus in sets of 209 and 159 peptides, respectively (Figure S1A,B). Controls were performed in which the antibodies specific against the interaction partner to be tested, that is, anti‐FliC antibodies to the DnaK SPOT membrane and vice versa, were directly applied to each membrane array without prior interaction partner incubation (Figure S1A,B). Antibodies were applied at a dilution of 1:1000 in PBS. Visualisation of bound antibodies was achieved via incubation with anti‐rabbit secondary antibodies fused to alkaline phosphatase at 1:10,000 dilution (ThermoFisher, Munich, Germany) (Molecular Probes, Eugene, OR). After alkaline phosphate‐mediated chromogenic reactions, the membranes were photographed and the relative intensity of each labelled peptide analysed using the ImageJ Region of Interest (ROI) Manager software (Schindelin et al. 2012). The same defined area selected with the smaller spot of interest, a ROI, was used for the multiple selection of all positive spots and a background empty space spot for intensity measurements. The relative intensity derived from the background selection was subtracted from each individual spot intensity value. Peptides giving rise to weak chromogenic reaction in the control membranes that resulted in comparable intensity values to those encountered in the assayed membranes were excluded from the analysis. The resulting labelled peptides are reported in the Table S2. Only peptides with twofold intensity over the membrane background were considered for the model analysis and are presented in Table 2.

### 2.6 Immunoprecipitation of DnaK Using Anti‐DnaK IgG

*Pseudomonas aeruginosa* PA14 was grown in 200 mL LB broth at 37°C with 120 rpm agitation for 24 h. Cell cultures were diluted with 200 mL PBS and centrifuged at 9000 *g* for 20 min in a Beckman Coulter Allegra 25R centrifuge (Beckman Coulter, Krefeld, Germany). The cell pellets were washed in the same volume of PBS and centrifuged as described before. The pellets were resuspended in 20 mL of lysis buffer (50 mM Tris–HCl pH 7.5, 100 mM NaCl and 1 mM DTT supplemented with a protease inhibitor mixture (Sigma‐Aldrich, Taufkirchen, Germany). The cells were lysed via passage through a French press cell at 18,000 psi for four times. The cell free extracts were clarified by low‐speed centrifugation 5000 *g* for 5 min twice. The bacterial extracts were stored at −80°C. Proteins concentrations were determined using their absorbance at 260 and 280 nm as described (Stoscheck 1990). The co‐immunoprecipitation (IP) was performed using Dynabeads Protein A (Invitrogen) in combination with a DynaMag magnet following the Dynabeads magnetic separation technology according to the manufacturer's recommendations. Briefly, 10 μg of purified IgG anti DnaK (Borrero‐de Acuña et al. 2015) were incubated and rotated for 15 min with Dynabeads Protein A in 200 μL of PBS with 0.02% Tween20. The magnetic bead–antibody complexes were magnetically separated, washed and resuspended in 200 μL PBS/Tween. The sample with the antigen consisting of a cell lysate diluted to a protein concentration of 1.4 mg/mL was incubated with the beads/antibody complex for 10 min under constant rotation. The Dynabeads‐antibody–antigen complex was washed three times with PBS/Tween, resuspended in 100 μL and transferred to a clean tube where the supernatant was removed. Twenty microliter elution buffer (100 mM citrate pH 2.5) was added, mixed gently and incubated under rotation for 2 min at room temperature to dissociate the complex from the Dynabeads. Then the supernatant containing the eluted antibody–antigen complex was transferred again to a clean tube and the pH of the eluate was adjusted by adding 100 μL of 1 M Tris pH 7.5. The sample was immediately stored at −80°C until the LC–MS–MS analysis was performed.

### 2.7 Liquid Chromatography Tandem Mass Spectrometry (LC–MS/MS)

The LC–MS/MS analyses were performed on a DionexUltiMate 3000 n‐RSLC system coupled with to an LTQ Orbitrap Velos mass spectrometer (Thermo Scientific, Germany). Protein samples were digested by adding trypsin at a protein/protease ratio of 50:1 and incubated at 37°C overnight. After evaporation of all fluid in a SpeedVac (Eppendorf, Hamburg, Germany), the peptides were solubilised in 3% acetonitrile (ACN) containing 0.2 trifluoroacetic acid (TFA). Peptides were loaded onto a C_18_ precolumn (3 μm RP18 beads, Acclaim, 75 μm by 20 mm; Dionex, Thermo Scientific, Germany) in 3% ACN containing 0.1% TFA, washed for 3 min in the same solution at a flow rate of 6 μL/min. Subsequently, the peptides were separated on a C_18_ analytical column (3 μm, Acclaim PepMap RSLC, 75 μm by 25 cm; Dionex) at a flow rate of 350 μL/min via a linear 120‐min gradient from 100% buffer A (0.1% formic acid in water) to 25% buffer B (99.9% acetonitrile with 0.1% formic acid), and subsequently a 50 min gradient from 25% buffer A to 80% buffer B. Chromeleon software (version 6.8; Dionex) coupled with the Xcalibur software suite (version 2.1; Thermo Scientific, Germany) was used to operate the LC system. The column resulting effluent was electrosprayed (PicoTip emitter needles; Thermo Fischer Omnilab, Germany) into the mass spectrometer (Orbitrap Velos Pro). The mass spectrometer was controlled employing the Xcalibur software in a data dependent mode allowing the automatic selection of 10 double‐ and triple‐charged peptides and their corresponding fragmentation, which was conducted using LTQ settings (minimum signal, 2000; isolation width, 4; normalised collision energy, 35; default charge state, 4; and activation time, 10 ms). Raw data were processed by means of the Proteome Discoverer program (version 1.4; Thermo Scientific) on a Mascot server (version 2.3.02; Matrix Science). The peptide database of the *P. aeruginosa* PA14 strain was downloaded from Swiss‐Prot (Bairoch and Apweiler 2000). Parameters employed to search for proteins were: enzyme, trypsin; maximum missed cleavages, 1; fixed modification, MMTS(C); variable modifications, oxidation (M); peptide tolerance, 10 ppm; and MS/MS tolerance, 0.4 Da. The results were evaluated and quantitated using Proteome Discoverer 1.4 (Orsburn 2021).

### 2.8 General Growth Conditions for Microscopy Studies

*Pseudomonas* strains were grown overnight in 20 mL LB at 37°C and 120 rpm in 125 mL flasks. Cultures were diluted to an OD_600_ of 0.05 in 50 mL fresh medium in 250 mL flasks and incubated further at 120 or 140 rpm as indicated. Aliquots were obtained at different time points and different fixations protocols were used in parallel as indicated in the different microscopy studies performed.

### 2.9 Immunofluorescence Labelling, Light and Confocal Microscopy

For indirect immunofluorescence staining of DnaK and FliC, bacterial cultures were pre‐fixed by adding the same volume of a 6% paraformaldehyde solution in PBS and further incubated at room temperature for 20 min. No washing of the cells or shaking of the cultures were performed to avoid possible damage of the flagellar structures. Aliquots of 1.5 mL were centrifuged in an Eppendorf 5415R centrifuge for 3 min at 3500 rpm and the resulting cell pellets were resuspended in 100 μL of PBS. In parallel coverslips coated with a 0.01% poly‐l‐Lysine solution in water were prepared as previously described (Molinari 2016). Coverslips were first incubated with 50 μL of bacterial suspension and the bacteria were fixed to the glass with a 3% paraformaldehyde solution in PBS. The coverslips were transferred to a 24‐well plate, treated 20 min with a 10 mM glycine in PBS solution to quench the residual aldehyde groups that may bind the primary and secondary antibodies generating background and then washed with PBS. The immunostaining was performed at room temperature and coverslips were rinsed twice with PBS after each immunostaining step. Samples were permeabilised with 0.1% Triton X‐100 in PBS for 5 min, washed twice and incubated with blocking buffer (10% FBS in PBS) for 45 min before different parallel staining techniques were applied. Coverslips were incubated with the anti‐FliC or anti‐DnaK primary rabbit polyclonal antibodies diluted 1:100 in blocking buffer for 1 h. After washing, samples were incubated 45 min with the fluorescently‐labelled secondary antibodies (Thermo Fisher Scientific). Goat anti‐rabbit Alexa Fluor 488 and goat anti‐rabbit Alexa Fluor 564 were used for staining FliC and DnaK, respectively. After several washing steps, the coverslips were mounted using ProLong Gold antifade reagent (Thermo Fisher Scientific). Immunostainings using only one primary antibody were performed to stain FliC or DnaK independently or one labelling after the other for the double labelling of first FliC and secondly DnaK in the same sample. The immunostaining experiments, done in duplicates, have been repeated with at least three biological independent bacterial cultures. Samples were first analysed with an Axio Imager A1, AxioCam MRm camera, and ZEN blue microscopy software (Zeiss, Oberkochen; Germany). In addition, samples were also analysed with a Leica TCS SP5 laser scanning confocal microscope (Leica Microsystems, Germany) and the images analysis was performed using Fiji/ImageJ (Schindelin et al. 2012).

### 2.10 Immuno‐Labelling of Bacteria for Transmission (Negative Staining) and Scanning Electron Microscopy

Bacteria were fixed with 2% formaldehyde in the growth medium, kept overnight at 7°C, pelleted and resuspended in PBS containing 10 mM glycine and left for 15 min. After centrifugation the pellet was resuspended in 250 μL of a 1:25 dilution of the protein A‐sepharose purified DnaK IgG antibodies and incubated for 1 h at 37°C. After washing twice with PBS bacteria were incubated with a 1:10 dilution of protein A/G coated gold nanoparticles (15 nm in diameter) in PBS containing 0.5% polyethylene glycol 20,000 for 30 min at 37°C. This step allowed for the visualisation of the bound DnaK antibodies. After washing twice with PBS, samples were washed with TE buffer (20 mM TRIS, 2 mM EDTA, pH 7.0). After washing steps with PBS and TE buffer samples were adsorbed onto carbon sputtered butvar‐coated copper grids (300 mesh) and observed in a Zeiss Merlin field emission scanning electron microscope (FESEM) at an acceleration voltage of 5 kV applying the high‐efficiency SE‐detector and in lens SE‐detector in a 82:18 ratio. For observation in a TEM, samples were negatively stained with 2% uranyl acetate and observed in a Zeiss TEM910 at an acceleration voltage of 80 kV. Contrast and brightness were adjusted using the software program Adobe Photoshop CS5.

### 2.11 Immuno‐Labelling on Ultrathin Sections

Ultrathin sections were cut with a diamond knife and collected onto butvar‐coated 300 mesh nickel grids and incubated overnight at 7°C in a 1:10 dilution of the protein A‐sepharose affinity‐purified specific anti‐IgG antibodies against DnaK or FliC. After intensive washing with PBS, bound antibodies were made visible by incubating with protein A/G‐conjugated with gold nanoparticles (PAG) 15 nm in size (1:75 dilution of the stock solution) for 30 min at room temperature. After washing with PBS containing 0.1% Tween 100, washing with TE‐buffer and distilled water followed, sections were counter‐stained with 4% aqueous uranyl acetate for 1 min and lead citrate for 30 s. Samples were then examined in a TEM910 transmission electron microscope (Carl Zeiss, Oberkochen, Germany) at an acceleration voltage of 80 kV. Images were recorded digitally at calibrated magnifications with a Slow‐Scan CCD‐Camera (ProScan, 1024×1024, Scheuring, Germany) with ITEM‐Software (Olympus Soft Imaging Solutions, Münster, Germany). Contrast and brightness were adjusted with Adobe Photoshop CS5.

### 2.12 Swimming Motility Assays

The swimming motility was tested as previously described (Rashid and Kornberg 2000). For that purpose, petri dishes supplemented with 1% (wt/vol) tryptone, 0.5% NaCl, 0.3% (wt/vol) agarose medium were prepared. Strains were grown aerobically overnight and a 1 μL from each culture was point inoculated in the middle. The plates were incubated under oxygen rich conditions at 37°C. Swimming motility halos from each strain were photographed after 24 h.

### 2.13 Modelling of DnaK–FliC Interactions and Molecular Dynamics Simulations

In order to obtain DnaK–FliC complex structures a docking ensemble of 97,602 poses was produced using RosettaDock based on the EMBL AlphaFold predictions for DnaK (Q02FR1) and FliC (A0A0H2Z7X1). The docking ensemble was then analysed with regards to the distance between the peptides from both proteins, thus, the distance between the FliC peptides and DnaK, as well as the distance between the DnaK peptides and FliC. This was done via a custom written python script using the packages SciPy (Virtanen et al. 2020), NumPy (Harris et al. 2020), and the Biotite library (Kunzmann and Hamacher 2018; Kunzmann et al. 2023), as well as matplotlib (Hunter 2007). The protein visualisations were created with PyMol (Schrödinger and DeLano 2020).

To obtain the structure of the DnaK in complex with FliC multimer modelling was used in AlphaFold version 2.3. Here structures from the PDB until May the 24th 2022 were considered as templates. An ensemble of 78 models was generated from three AlphaFold multimer runs. All of these structures were loaded and aligned on the FliC protein in PyMol (Schrödinger and DeLano 2020) and the pose with the most connections between DnaK and the FliC D2/D3 region was found based on spatial contacts. All figures were created using PyMol (Páll et al. 2020; Schrödinger and DeLano 2020).

Molecular dynamics (MD) simulations of the selected multimer modelling were performed in GROMACS (Páll et al. 2020) by using the CHARMM36 force field (Huang and MacKerell Jr 2013). After placing the molecule inside a dodecahedron with periodic boundary conditions, ions were added to neutralise the overall charge. After equilibration for 100 ps in the NVT (*n*umber of particles, *v*olume, *t*emperature) and then the NPT (*n*umber of particles, *p*ressure, *t*emperature) ensembles, the production run was performed for 500 ns at 300 K and 1 bar, following the protocol for MD simulations as described in Bogetti et al. (2019). To analyse the stability of the MD simulation, the root mean square deviation (RMSD)

$$\text{RMSD}\left(t\right)=\sqrt{\frac{1}{N}\sum_{i=1}^{N}{\left(r_{i}\left(t\right)-r_{i}\left(0\right)\right)}^{2}}$$

measuring the average deviation of each atom from a reference was used. Furthermore, the radius of gyration *R*_*g*_

$$R_{g}=\sqrt{\frac{\sum_{i}m_{i}{\left|\vec{r_{i}}\right|}^{2}}{\sum_{i}m_{i}}}$$

measuring the root mean square distance from the centre of mass, was used to analyse conformational changes. To analyse the agreement of the protein–protein complexes with the found peptides, the minimum distance ∆r between the peptide region and the partner protein was used to indicate spatial proximity. Again, a custom python script based on the same package as before, but mostly biotite was used.

### 2.14 In Vitro DnaK Holdase Activity Assay

SOD1_bar_ mutant G41D (SOD1_bar_‐G41D) was selected from a library of mutants previously built and purified accordingly to the established protocol (Gnutt et al. 2019). Frozen aliquots of the protein were left to equilibrate at room temperature for 30 min and further diluted in 20 mM acetate (Sigma), 20 mM MES (Sigma), 20 mM sodium phosphate (Sigma) buffer, pH 7 to a final concentration of 10 μΜ, either supplemented with 20 μΜ DnaK and/or 2 mM ATP (Sigma) or 2 mM AMP‐PNP (Sigma). For each solution, pH values were re‐adjusted to the desired ones prior to the measurement with concentrated solutions of HCl (12 M) and NaOH (10 M) (Mettler Toledo pH meter FiveEasy).

The SOD1_bar_‐G41D solution was incubated with DnaK and/or adenosine nucleotides for 32 and 24 min at pH 7 and 6, respectively. Every 8 min, 14 μL of sample were placed between a glass bottom dish (FluoroDish, WPI) and coverslip (VWR), attached to one another through a 120‐μm imaging spacer (Secure Seal, Sigma). Images of AcGFP1(D) (497–527 nm) and mCherry (A) (581–679 nm) emission were acquired by two CCD cameras assembled into a Zeiss AxioObserver Z1 widefield fluorescence microscope, every two frames per second. AcGFP1 was excited by a 470 nm LED light with similar exposure time for each measurement. These data were assessed using ImageJ (Schneider, Rasband, and Eliceiri 2012) and Matlab software (MATLAB 2022). A region of interest (ROI) of the same pixel size was drawn on each picture for both ACGFP and mCherry channels and averaged to determine the D/A (or FRET ratio) as a function of time. For initial FRET ratio determination, pictures were taken during 12 s at RT and final values were averaged from the last 3 s.

Temperature‐sensitive rhodamine B (Rho‐B, Carl Roth) fluorescent dye (100 μM) was used to calibrate the laser heating jumps as previously described (Büning et al. 2017). The temperature (T) versus time plot (Figure 6A, left) was built by converting the normalised Rho‐B signal decrease as a function of temperature using the equation

(1) $$I_{\mathrm{Rho}-B}\;\left(T\right)=1.652-0.03238\;T+0.0002082\;T^{2}$$

(Equation 1). The size of the first 10 temperature jumps was then averaged resulting in a final value of 2.3 ± 0.3 K.

SOD1_bar_‐G41D exhibits a two‐state folding behaviour, transiting between the native (N) and unfolded (U) states in a reversible manner (Gnutt et al. 2019). For determination of melting temperature (*T*_*m*_) and modified standard state free energy of folding at RT or 310.15 K (Δ*G*_*f*_^0’^), we adopted the ‘thermodynamics from kinetics approach’ developed by Gruebele and co‐workers (Girdhar et al. 2011). For each unfolding experiment, individual unfolding kinetics shown as the donor to acceptor difference, D‐αA ($\alpha =\frac{D_{\left(T_{0}\right)}}{A_{\left(T_{0}\right)}}$, where *T*_0_ is the signal at time = 0 s for each T‐jump), were plotted as a function of time (*t*) and fitted to a single exponential function (

(2) $$D\left(t\right)-\mathrm{\alpha A}\left(t\right)=A_{t=0}+A∙\left(1-e^{\left[-k∙t\right]}\right)$$

(Equation 2), where *A*_*t*=0_ and *A* are the initial and maximum resolved unfolding amplitudes, respectively and *k* the rate constant) (Figure 6D). A (or *D*‐*αA*_MAX_) values were further plotted as a function of temperature (Figure 6D), from which *T*_*m*_ values were extracted after fitting to the following Equation (3) (Girdhar et al. 2011):

(3) $$D\left(T\right)-\mathrm{\alpha A}\left(T\right)=\frac{-g^{\left(1\right)}\cdot ∆T\cdot T_{m}}{R\cdot {\left(T-\frac{∆T}{2}\right)}^{2}}\cdot \left[A_{0}+m_{A}\cdot \left(T-T_{m}\right)\right]\cdot \frac{\exp\left[-g^{\left(1\right)}\cdot \frac{\left(T-\frac{∆T}{2}-T_{m}\right)}{R\cdot \left(T-\frac{∆T}{2}\right)}\right]}{{\left(1+\exp\left[-g^{\left(1\right)}\cdot \frac{\left(T-\frac{∆T}{2}-T_{m}\right)}{R\cdot \left(T-\frac{∆T}{2}\right)}\right]\right)}^{2}}$$

where *A*_0_ and *m*_*A*_ define the baseline of *A* (*m*_*A*_ was restrained to 0), Δ*T* refers to the size of the temperature jump (here set to 2.3 K) and $g^{\left(1\right)}$ is the cooperativity parameter. Δ*G*_*f*_^0’^ was calculate using the following linear relation:

(4) $$\Delta G_{f}^{0’}=g^{\left(1\right)}\cdot \left(T–T_{m}\right)$$

(Equation 4). All the above assumptions were made by constraining the change in the heat capacity of unfolding (Δ*C*_*p*_) to 0 (Girdhar et al. 2011).

Folding rates (*k*_*f*_) at 310 K (~37°C) were calculated via the following relation between the folding equilibrium constant *K*_*f*_ and the computed *k* at 310 K (Equation 5) (Guo, Xu, and Gruebele 2012):

(5) $$k_{f}=k\cdot f_{\text{Native}}↔k_{f}=\frac{k\cdot K_{f}}{\left(1+K_{f}\right)}$$

*K*_*f*_ (*T* = 310 K) was determined via ${∆G}_{f}^{0’}\left(T\right)$ = − *R*·*T*·ln *K*_*f*_(*T*). Average and standard deviation values for all fitted parameters (*T*_*m*_, *g* ^(*1*)^, Δ*G*_*f*_^0’^ and *k*_*f*_) at the various conditions are shown in Table S3.

It should be noted that in vitro DnaK thermal unfolding at pH 6.5 is reported to involve four states, with three transitions characterised by *T*_*m*_ values of 319.55, 330.15 and 346.55 K, respectively (Montgomery et al. 1993). *T*_*m*_ of SOD1_bar_‐G41D when supplemented with DnaK is on average 308.1 ± 0.1 K and therefore the likelihood of nonspecific interactions between the (partially) unfolded states of both DnaK and SOD1_bar_‐G41D are low.

## 3 Results

### 3.1 FliC Is a Major Binding Partner of the Chaperone DnaK in *P. aeruginosa* PA14

We previously discovered a triple FliC–DnaK–NirS complex in the periplasm of *P. aeruginosa* cells grown under anaerobic conditions and respiring by denitrification (Borrero‐de Acuña et al. 2015). Furthermore, affinity chromatography‐based purification assays revealed co‐elution of the recombinant FliC and DnaK proteins (Borrero‐de Acuña et al. 2015). In order to investigate a potential function of the FliC–DnaK complex in cells grown in aerobic conditions, proteins cross‐linked to DnaK by formaldehyde treatment of *P. aeruginosa* PA14 cells grown under aerobic conditions were captured by co‐immunoprecipitation using an anti‐DnaK polyclonal antibody (IP) of whole *P. aeruginosa* cell lysate samples. The composition of the precipitated protein complexes was analysed by LC–MS/MS. Table 1 shows the experimentally obtained DnaK interaction partners from *P. aeruginosa* classified by their relative abundance. The value reflects the relative degree of abundance to which each interaction partner is present in the sample. Higher values indicate a strong quantitative association of the corresponding prey protein to its bait DnaK. The number of unique peptides and the corresponding percentage of protein sequence coverage are also shown. Only proteins identified by at least two unique peptides were considered as interacting prey proteins.

**TABLE 1:** Interaction partners of DnaK elucidated by Co‐IP/LC–MS–MS.

Protein name	Ref. number	Coverage	UP	Relative abundance^a^
Flagellin type B FliC^b^	PA14_50290	53%	24	1.736E8
Chaperonin GroEL^c^	PA14_57010	44%	21	9.076E6
Outer membrane lipoprotein OprI precursor^d^	PA2853/ND	39%	4	3.888E6
Major porin and structural outer membrane porin OprF precursor^d^	PA14_41570	21%	4	3.721E6
Dihydrolipoamide acetyltransferase AceF^e^	PA14_66310	22%	2	1.348E6
Dihydrolipoamide dehydrogenase Lpd^e^	PA14_43970	15%	5	1.154E6
Hypothetical protein PA3309 UspK^f^	PA14_21220	14%	2	6.401E5
Outer membrane protein OprG precursor^d^	PA14_11270	16%	2	5.518E5

Abbreviation: UP, unique peptides.

^a^ Relative abundance was quantified by averaging out the area of the three most prominent peptides of each protein. Proteins are ordered by relative abundance. Clusters of Orthologous Groups (COGs).

^b^ Motility.

^c^ Posttranslational modification, protein turnover and chaperones.

^d^ Cell wall, membrane and envelope biogenesis.

^e^ Energy production and conversion.

^f^ Universal stress.

A very limited number of proteins co‐precipitated with DnaK, which is consistent with a high specificity of the experimental approach. FliC, the monomer flagellin type B that polymerises to form the filament of the flagellum, was found to be the most abundant interaction partner (100‐fold enriched compared its regular abundance in the proteome). The GroEL chaperonin, which is part of the molecular chaperone complex GroEL‐ES that helps other proteins fold correctly in the cell (Hayer‐Hartl, Bracher, and Hartl 2016), was found as an interactor of DnaK, although to a lesser extent. Apart from small contaminating amounts of various ribosomal subunits (not listed), several major protein constituents of the outer membrane, namely porins OprI, OprF and OprG, were co‐precipitated with DnaK. A close interaction of these Opr proteins with each other has been observed before by crosslinking‐based interactomics (Navare et al. 2015; Chevalier et al. 2017; Cassin and Tseng 2019). Subsequent findings suggested a close interaction of DnaK with the outer membrane, probably on the extracellular face of the membrane. Moreover, two subunits of the central metabolic pyruvate dehydrogenase complex, the dihydrolipoamide acetyltransferase AceF (E2) and dihydrolipoamide dehydrogenase Lpd (L3) were found attached to DnaK. Interestingly, the pyruvate dehydrogenase subunits and the Opr proteins have the same bound lipid in common. The joint secretion of DnaK and pyruvate dehydrogenase during the stationary phase of *Bacillus subtilis* has been previously observed (Yang et al. 2011; Wang et al. 2012). Both proteins have also been identified as two out of four immunogenic proteins in mini‐pigs when challenged with planktonic and biofilm forms of *Streptococcus suis*, which is also indicative of their surface localisation (Wang et al. 2012). Finally, the universal stress protein UspK involved in the stringent response during nutrient deprivation, was found as DnaK interaction partner (Eschbach et al. 2004). By means of immunoprecipitation, the NEF GrpE protein and the DnaJ, which are crucial components of the classical DnaK/DnaJ/GrpE chaperone folding system (Brehmer et al. 2004), were not identified as a DnaK interaction partners in aerobically grown *P. aeruginosa* cells. These results are consistent with an extracellular location and function of DnaK, in addition to its classical intracellular ATP‐dependent chaperone role, and its additional periplasmic role in the FliC–DnaK–NirS interaction.

### 3.2 Molecular Basis of the DnaK–FliC Interaction

To further characterise the DnaK–FliC interaction, a SPOT‐membrane peptide array was performed (Frank and Overwin 1996). In this array, each membrane spot contained a synthetic peptide of 15 amino acids with an overlap of 12 amino acids with the peptide of the adjacent spot, shifting the sequence each time by 3 amino acids. The array spanned the entire *P. aeruginosa* DnaK and FliC protein sequences, from the N‐ to the C‐terminus in sets of 209 and 159 peptides, respectively (Figure S1A,B, top). Detection of the interacting peptide was performed using purified recombinant *P. aeruginosa* DnaK for the FliC SPOT‐membrane and purified recombinant *P. aeruginosa* FliC for the DnaK SPOT membrane. Bound proteins (DnaK or FliC) were detected with appropriate antibodies. Controls were the antibodies applied directly to each membrane, that is, anti‐FliC antibodies to the DnaK SPOT membrane and vice versa, without prior incubation with purified DnaK or FliC proteins (Figure S1A,B, bottom). After alkaline phosphatase‐mediated chromogenic reaction, the membranes were photographed and the relative intensities were measured for each peptide using ImageJ ROI Manager and further analysed (see Section 2). Spots with intensity values above 2 were used for the mapping of the DnaK–FliC interaction sites and are listed in Table 2. The list of all peptides chromogenically detected to some extent are reported in Table S2.

**TABLE 2:** Peptides identified by Spot membrane array and their intensities.

Peptide Nr.	Peptide sequence	Intensity^a^	Domain^b^
DnaK			
H14	QGDALVHATRKMITE	3.336	SBD
E18	IHEVILVGGQTRMPL	2.234	NBD (ATPase)
H13	ARNQGDALVHATRKM	2.225	SBD
E20	VGGQTRMPLVQKTVA	2.224	NBD (ATPase)
FliC			
B18	TRISDTTTFGGRKLL	5.065	D1
A16	AGLQISNRLSNQISG	4.172	D1
E22	TGYVQLNSPTAYSVS	3.554	D2
C20	VGGGQVKNIAIAAGD	3.05	D3
E21	TVVTGYVQLNSPTAY	2.873	D2
D20	LADQLNSNSSKLGIT	2.854	D2
A9	LNTSLQRLTTGYRIN	2.506	D0
A10	SLQRLTTGYRINSAK	2.396	D0
E20	ATTTVVTGYVQLNSP	2.363	D2
F19	QRADLGAVQNRFKNT	2.339	D1
F22	NRFKNTIDNLTNISE	2.302	D1
D5	IPNLSARARTVFTAD	2.222	D3

^a^ Peptides intensities were measured using ImageJ ROI Manager. Only peptides with twofold intensity over the membrane background are shown and considered in the analysis.

^b^ The protein domains to which each peptide correspond are displayed. SBD, substrate binding domain; NBD, nucleotide‐binding domain; D0–D3 domains.

In Figure 1 the structure for the two respective protein primary and tertiary structures of DnaK in A and C and FliC in B and D are illustrated. The structures for DnaK (Q02FR1) and FliC (A0A0H2Z7X1) were obtained from the EMBL (AlphaFold Protein Structure database) (Jumper et al. 2021; Varadi et al. 2022). The major functional domains of the DnaK molecular chaperone are the nucleotide‐binding domain (NBD) connected by a flexible linker to the substrate binding domain (SBD) (Figure 1A). The NBD is responsible for the ATP hydrolysis essential for the foldase activity, the active transition of misfolded proteins back to their native conformation executed by the SBD. The SBD is also able to perform an ATP‐independent holdase activity which consists of the holding of the substrate and prevention of aggregate formation (Wu et al. 2012; Lu and Swartz 2016; Rutledge, Choy, and Duennwald 2022). The SBD consists of a β‐sandwich region and the α‐helical lid, which is covering the substrate binding cleft (Figure 1A). The FliC monomeric structure is subdivided into four domains: D0–D3 (Figure 1B) (Wang et al. 2017). During polymerisation, 11 protofilaments are assembled directed upright with interactions between domains D0 and D1 in the filament inner core (FIC) of the flagellum (Wang et al. 2017). Conversely, the D2 and D3 domains are exposed on the surface (filament outer core; FOC) and might be prone to interactions with DnaK in this polymeric state (Figure 1B). In Figure 1C,D interacting peptides encountered in the SPOT‐membrane array for both proteins are highlighted in magenta in both proteins.

Using the structural information collected from SPOT membrane assays (binding peptides from Table 2), we investigated the basis for the DnaK chaperone interaction with FliC using a in silico structural modelling approach. For this purpose, we performed a global docking analysis as an intermediate step (Leman et al. 2020). In this step, a large ensemble of conformations (97 k) was generated and filtered by analysing for spatial proximity of the according peptide sequences to the respective partner protein. Taking peptide H14 from DnaK as an example, the position in the ensemble in which H14 of DnaK showed the shortest distance to any atom of FliC was searched. The according minimal distance positions are illustrated for all DnaK peptides in Figure S2A and for all FliC peptides in Figure S2B. A wide range of conformations was found indicating that no conformation minimises all the distances at the same time. Therefore, we also used AlphaFold multimer modelling (Evans et al. 2021) to generate structures where both proteins are co‐folded using AI going beyond the capabilities of statical molecular docking. A total of 78 models showed overall good scores (ensemble illustrated in Figure S3). It is noteworthy to consider that AlphaFold is trained on the structural information available on all experimentally resolved protein structures in a folded state (Jumper et al. 2021), Therefore, potential interactions occurring between the DnaK SBD and an ensemble of intermediate unfolded states of FliC are not captured by this artificial intelligence (AI)‐aided computational analysis. Figure S2A shows that both DnaK peptides located in the α‐helical lid of its SBD (H13 and H14) interact with the D0 of FliC, which in other bacteria like *Salmonella* spp. appears to be its most disordered domain (Aizawa et al. 1990; Minamino et al. 2021). Interestingly, although peptides from the DnaK NBD are presumed to interact exclusively with ATP and not to peptide substrates, it was observed that peptides E18 and E20 of this region bind with the D1 of FliC. Among all interactions modelled in Figure S2B for FliC peptides, a pattern became significant. D2 and D3 domains of FliC appear to interact preferentially with the NBD of DnaK. This kind of DnaK‐FliC docking arrangement, that is, maximising the contacts between D2/D3 and DnaK, will be further examined in the following sections. Likewise, the D1 domain appears to be prone to interact with the NBD. Conversely, the D0 of FliC binds preferably to the α‐helical lid of the SBD of DnaK. Thus, interactions between the lower stretches of FliC, normally more disordered, seem to be biased towards the chaperone‐acting SBD of DnaK. In contrast, higher stretches of FliC, constituting the outwardly exposed regions in a reconstituted filament, are inclined to interact with the NBD of DnaK.

The array of interactions encountered here may be targeted for the design of antagonist peptides interfering with the binding of DnaK–FliC, which can be administered in small molecule cocktails for preventing *P. aeruginosa* colonisation and infection (Lu et al. 2020).

### 3.3 Molecular Dynamics Simulations of the DnaK–FliC Interaction

Next, a 1000‐ns molecular dynamics simulation was performed with GROMACS (Berendsen, Spoel, and Drunen 1995; Lindahl, Hess, and Van Der Spoel 2001; Van Der Spoel et al. 2005; Hess et al. 2008; Pronk et al. 2013; Abraham et al. 2015; Páll et al. 2015) following an updated protocol by J.A. Lemkul (Huang et al. 2017; Lemkul 2019). The results of this simulation are illustrated in Figure S4. Two quantities used to characterise dynamics trajectories, that is, root mean square deviation (RMSD) as well as radius of gyration (R_g_/Radius) are visualised in Figure S4A. These two quantities describe the average deviation from the starting pose (RMSD), as well as the radius, by fitting the protein into a sphere (R_g_), respectively.

As can be seen in Figure S4A, conformational change was observed at around 400 ns, where both quantities underwent sharp changes in trajectory. The RMSD appeared to be stable afterwards, while the radius of gyration started to change again at the end of the simulation. It was observed that a stable cluster was reached (the semi‐circular region of high density on the right lower hand side) when a two‐dimensional histogram of both states was plotted (Figure S4C), In addition, the scatter plot (Figure S4D) showed that the corresponding region was located at the end of the simulation. This showed that the simulation stabilised in both quantities, RMSD as well radius of gyration, indicating that this may indeed be a native protein structure, as native protein structures appear to be consistently more stable in simulations (Karaca, Prévost, and Sacquin‐Mora 2022). Furthermore, the percentage of initial contacts for only the atoms in the initial interface between DnaK–FliC in the input model was visualised in Figure S4B, as well as the percentage of absolute contacts between both proteins. It was clearly visible that the initial interface contacts decline, while the dimer contacts increased until around 500 ns and subsequently stabilised. In total, the generated 1000 ns trajectory showed a stabilised dimer that differed from the input dimer generated by AlphaFold. Figure S5 shows a series of snapshots illustrating the time evolution towards this new conformation. Here the visualisation stops at 500 ns as there is no observable change in the protein–protein interface in the last 500 ns of the simulation, as also indicated by the stabilisation in RMSD and Rg from 500 ns on. Furthermore, in Videos S1–S4, the entire simulation is presented in different visualisations and viewpoints. Throughout this time evolution (Figure S5 and Videos S1–S4, the trajectory of the DnaK body pivoting over the FliC molecule by acquiring different conformations is observed. Initially, a stable DnaK–FliC protein complex is achieved by interactions at the interface of β‐strand of the NBD and the D2 and D3 domains of FliC. Subsequently, the NBD of DnaK swings downwards allowing new interactions to occur between the D1 of FliC and other regions of the NBD as well as with the β‐sandwich of the SBD. The interaction trajectory is finalised when this swinging of the DnaK body leads to the interaction between its α‐helical lid and the D0 of FliC.

Overall, computational analysis revealed a dynamic protein–protein interaction interplay, in which several DnaK–FliC conformations are acquired. Most likely, this reflects the dynamism governing this protein complex in vivo, where cytoplasmic and extracellular interactions must be coordinated with the unfolding process during export.

Previously, the involvement of the peptides found by SPOT‐membrane were not analysed as a function of the interaction trajectory. To this end, the absolute number of contacts between FliC peptides and DnaK was plotted over time in Figure S6A–D and likewise the absolute number of contacts between DnaK peptides and FliC was plotted over time in Figure S6E. The number of contacts for almost all of the FliC peptides undergoes a substantial rise starting from around 400 ns (Figure S6A–D) indicating multiple likely interactions. Nevertheless, certain peptides from FliC present more contacts at earlier time points. DnaK peptides H14 and H13 (both present in the SBD; Figure S6E) appear to present contacts after 400 ns. Thus, the peptides of DnaK belonging to the NBD, which were found by SPOT‐membrane do not seem to participate in the produced trajectory over time. Nevertheless, the SPOT‐membrane peptides that do interact seem to engage in the order indicated by the molecular dynamics analysis. The first FliC peptides to display contacts with the DnaK molecule during the course of the analysis appertain to the upper domains of FliC (D2/3), matching the events of Figure S3. Progressively, peptides of the D1 domain pop up before and precisely after 400 ns. In the last stages, peptides from the D0 domain of FliC and simultaneously those of the α‐helical lid of the DnaK SBD present stabilising contacts, indicating the events occurring at the latest time points of the calculated trajectory.

The absolute number of contacts between FliC peptides and DnaK and vice versa was plotted over time and described in Figure S6A–E. The trajectory observed by molecular dynamics for DnaK‐FliC interactions tends to match the timing of transient contacts with the different regions of each protein. In conclusion, the dynamic swinging over time of the DnaK, in which the NBD establishes initial contacts with the D2/D3 domains of FliC to subsequently rotate upon itself allowing new interactions between the SBD with the lower stretches (D1 and finally D0) of the FliC structure seems to be supported by this computational analysis.

### 3.4 Extracellular and Intracellular Localisation of the DnaK Chaperone

After the determination of certain aspects of molecular nature of the DnaK–FliC interaction, the question of the dynamics of cellular location of the complex arose, as FliC is the major component of the extracellular filament of the flagellum. For this, *P. aeruginosa* PA14 cells were investigated by light, confocal and electron microscopy, using different immunolabelling approaches with anti‐DnaK or anti‐FliC antibodies.

**FIGURE 2:**
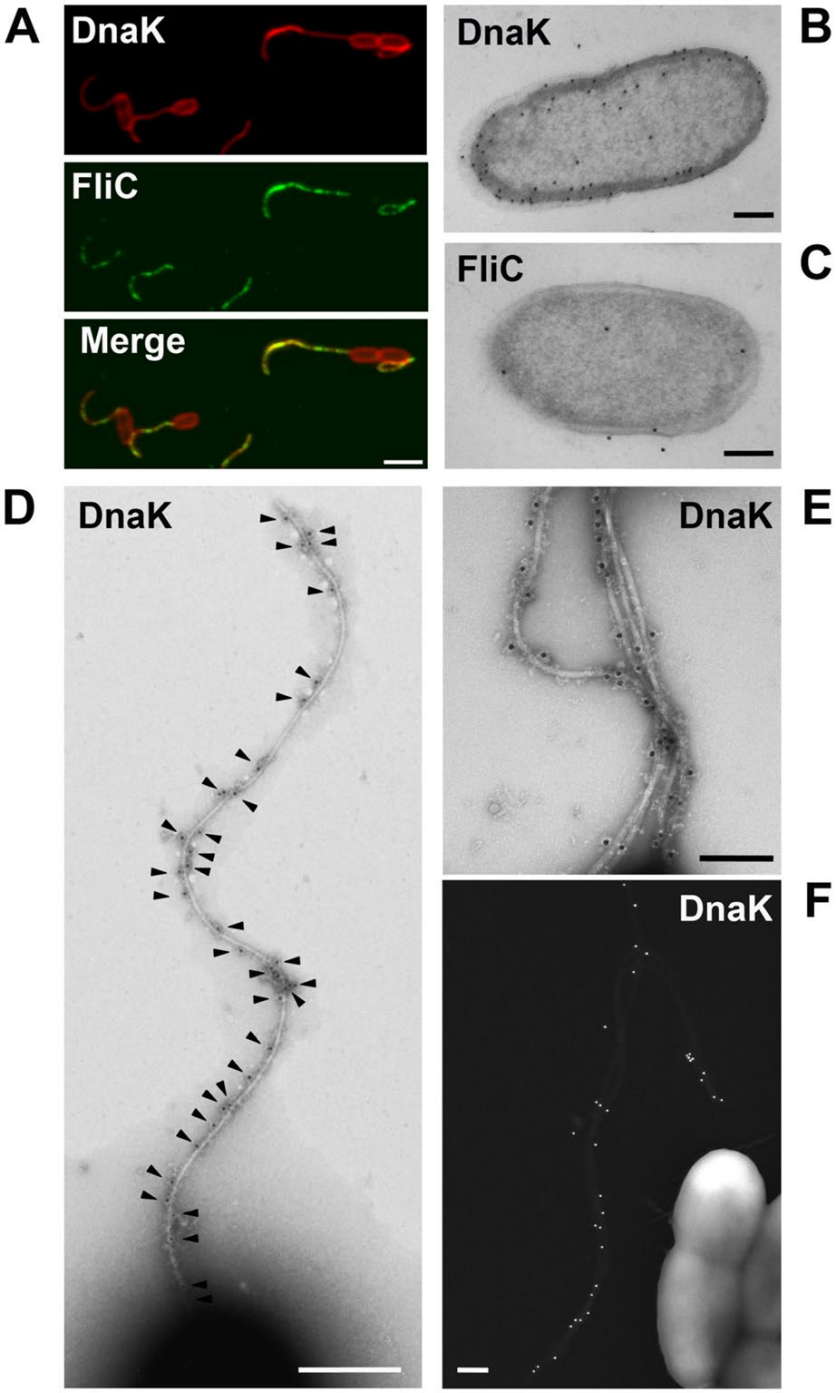
Extracellular and intracellular localisation of DnaK and FliC in *Pseudomonas aeruginosa* PA14. The co‐localisation analysis of DnaK and FliC is showed in (A) and was assessed by double immunofluorescence staining and confocal fluorescence imaging. DnaK (in red) was detected in the bacterial cells and outside in the flagella (top). FliC (in green) is present only in the flagellum (centre). The merge image (bottom) shows in yellow the co‐localisation of FliC and DnaK in the flagellum. The staining was performed after fixation and permeabilisation, firstly FliC was labelled with a rabbit anti‐FliC antibody and Alexa‐Fluor 488 conjugated goat anti‐rabbit antibody, then DnaK was labelled with a rabbit anti‐DnaK antibody followed by Alexa‐Fluor 568 conjugated goat anti‐rabbit antibody. Electron microscopy analysis of ultrathin sections of whole bacterial cells and immunogold labelling with gold nanoparticles of 15 nm size detected DnaK intracellularly mostly in the membrane area while only few gold particles are seen in the cytoplasm (B) and FliC scarcely present inside the bacteria (C). Immuno labelling of DnaK and negative staining for transmission (D, E) and scanning (F) electron microscopy was implemented to visualise the extracellular DnaK–FliC interaction. The chaperone was present distributed in all the flagellum length extension (black arrowheads in D) and at higher magnification, DnaK is observed associated to the flagellar filament contained in amorphous structures (E). Immunogold FESEM confirmed that, extracellularly, DnaK is localised coupled with the flagella and notably, revealed that is not present on the surface of the bacterial cells (F). Bars, 2 μm in (A), 200 nm in (B, C, E and F) and 500 nm in (D).

**FIGURE 3:**
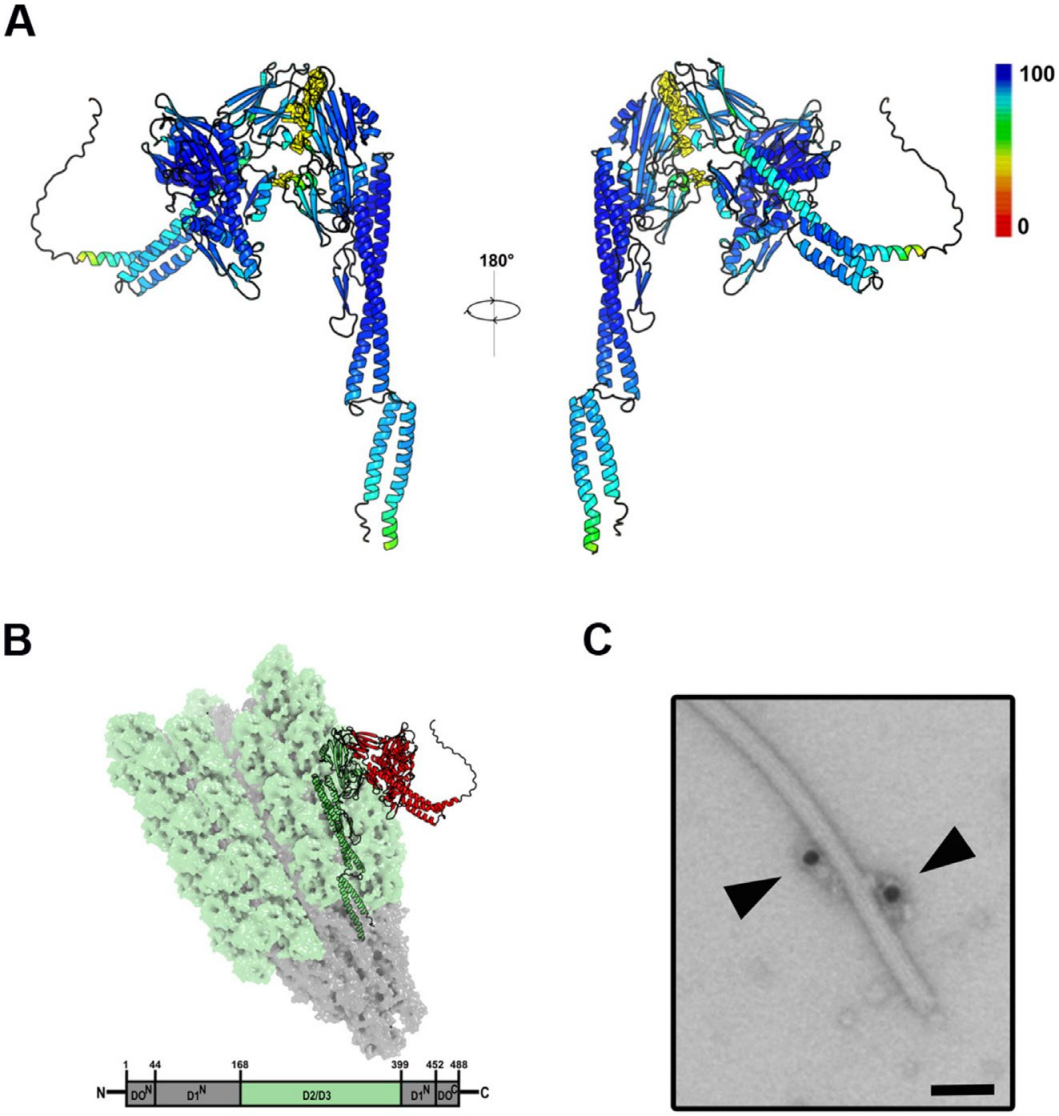
DnaK–FliC interaction. (A) In silico modelling of DnaK–FliC complex where a dimer of DnaK–FliC generated through AlphaFold multimer modelling is visualised. The per residue pLDDT score showing the quality of the prediction is given by the colouring of the residues. (B) Reconstructed FliC filament with the model showed in (A) aligned on surface. In this illustration a FliC filament based on the previously resolved filament structure 5WK5 (Wang et al. 2017) is shown. Here the previously unresolved part of the FliC protein making up the filament (see Figure 1) is highlighted in hell green with the DnaK (red)–FliC (green) complex generated with AlphaFold multimer modelling aligned on the surface. (C) Immunogold‐DnaK particles associated to a FliC filament in negative stain EM as showed in Figure 3D,E, here at higher magnification and mirroring the model presented in (B). Bar, 50 nm.

Co‐localisation of DnaK and FliC was assessed by double immunofluorescence staining. First, FliC was labelled in green with a rabbit anti‐FliC antibody and Alexa‐Fluor 488 conjugated goat anti‐rabbit antibody, then DnaK was labelled in red with a rabbit anti‐DnaK antibody followed by Alexa‐Fluor 568 conjugated goat anti‐rabbit antibody. As expected, DnaK labelling (red) was predominately concentrated in the cytoplasmic and outer membrane region of the bacterial cells. Strikingly, however, DnaK was also found to be extracellularly associated with the flagellum (Figures 2A top and S7A). Anti‐FliC antibodies detected the protein (green) exclusively as part of the filament of the flagellum (Figures 2A center and S7A). The merged images (Figures 3A bottom and S7A) confirmed the cellular localisation of DnaK (red cells) and indicated co‐localisation with FliC on the filament of the flagellum (yellow regions). Differential interference contrast (DIC) and fluorescence light microscopy, for the observation of bacterial cells labelled with only anti‐FliC antibody, confirmed that FliC was detected only extracellularly in the flagellum (Figure S7B).

In parallel studies, DnaK or FliC were detected with purified anti‐DnaK or anti‐FliC IgGs labelled with 15 nm gold nanoparticles (PAG15). In whole cell ultrathin sections of *P. aeruginosa*, labelled DnaK was restricted mostly to the envelope region of the cells (Figure 2B) which is in good agreement with the pattern and extent of labelling observed in immunofluorescence studies (Figure 2A). Very low numbers of gold particles were observed in the cytoplasm, indicating that in *P. aeruginosa* PA14 cells DnaK activity occurs mainly in the peripheral areas of the bacteria. Immunogold labelling of FliC revealed scarce protein abundance in the bacterial cells (Figure 2C).

Immunogold negative stain electron microscopy showed DnaK‐gold particles extracellularly associated with the flagellar filament along its entire length (Figure 2D,E). In addition, immunogold Field Emission Scanning Electron Microscopy (FESEM) clearly showed that extracellular DnaK was exclusively associated with the flagella and was not present on the surface of the bacterial cells (Figure 2F). This confirms that the DnaK shown by immunofluorescence to be associated with the bacterial cells, was protein intracellularly located in the membrane area.

### 3.5 Modelling the DnaK–Flagellum Association

To obtain a better understanding of the most likely DnaK–FliC interaction involved in the DnaK–flagellum association, a flagellum structural model was constructed based on previously resolved structures and the AlphaFold multimer modelling. The AlphaFold model where DnaK showed the most interactions to the FliC surface was selected and is shown in Figure 3A. In this model, the β‐strands of the DnaK‐NBD (ATPase) interact with the D2 and D3 regions of FliC. This sterically favoured protein complex configuration correlates with (i) the DnaK–FliC interactions shown in a manifold of the ensembles of Figure S5, and (ii) those interactions occurring during the first stages of the trajectory calculated by molecular dynamics simulations.

Subsequently, it was tested if this DnaK–FliC interaction (Figure 3A) was feasible to take place in a fully assembled flagellum scenario. Thus, the flagellar filament was reconstructed from the previously published partial resolved structure of the FliC filament (Abraham et al. 2015) and the AlphaFold model for FliC (A0A0H2Z7X1). The reconstruction was performed by aligning copies of FliC on the chains in the filament, identically to the filament generation in vivo (Páll et al. 2015). Next, the DnaK–FliC complex displaying most physical interactions (Figure 3A) was aligned to the surface of the filament (Figure 3B) showing no steric impediments or disturbances to the filament architecture. The model nicely mirrored the negative stain electron microscopy observations of DnaK gold particles found coupled with the flagellar filament (Figure 2D,E) and in Figure 3C showed at higher magnification. Thus, extracellular DnaK (shown in red in the Figure 3B model) is stably associated via its NBD domain with the surface exposed D2/D3 domains of the flagellins (shown in green in the same model) integrated in/constituting a fully assembled flagellum filament. This interaction seems to be sterically favoured in terms of number of contacts presented at the DnaK–FliC interface and also by the fact that it avoids disturbing the overall filament skeleton. Overall, multiple transient interactions have been observed in a 1:1 DnaK–FliC complex (shown in previous sections), whereas one likely conformation is favoured when DnaK is intercalated within the filament. This reflects the varying scenarios that these two proteins must undergo in the different cellular compartments in which they were found: cytoplasm, periplasm and extracellular environment.

The tendency of FliC is to auto‐polymerise through interactions of its D0 and D1 domains, which would lead to detrimental effects in the cytosol and periplasm. Thus, it is reasonable to assume that in these compartments the SBD of DnaK interacts with the D0 of FliC in order to prevent its polymerisation or aggregation. In contrast, the most stable interaction in the extracellular medium appears to be mediated by the NBD of DnaK—with no chaperoning action—and the D2 and D3 domains of FliC, which are naturally exposed to the filament surface. The DnaK and FliC proteins require to be translocated across the inner and outer membranes of the cell to reach the filament. Hence, it is possible that the different conformations presented in this work are indeed alternating states of this complex, which are formed depending on the demands of the export stage.

### 3.6 Interdependent Production and Localisation of DnaK and Assembled Flagellar Apparatus

**FIGURE 4:**
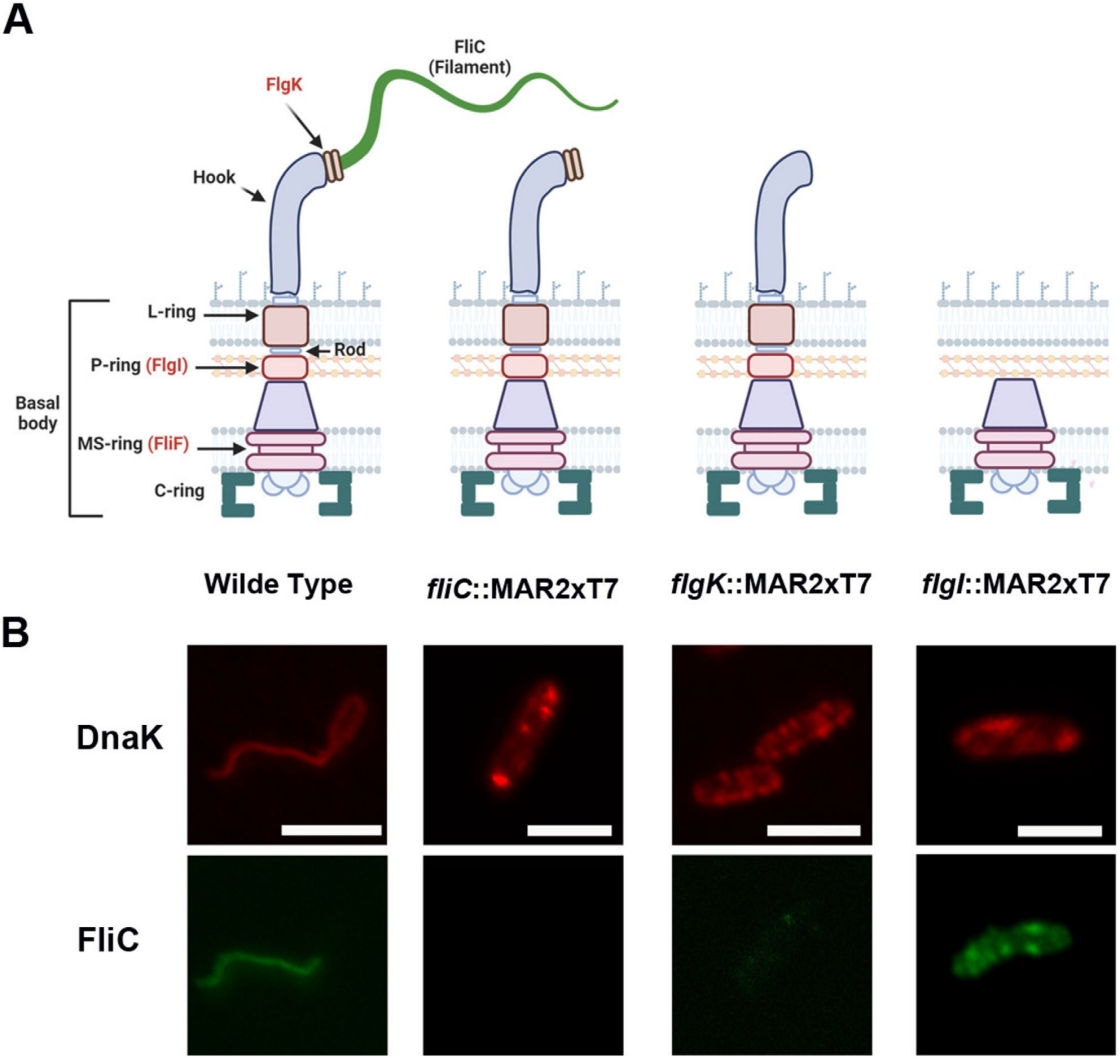
Localisation of DnaK and FliC in mutants deficient in the Type III‐like export. DnaK localisation and intracellular accumulation were assessed in transposon mutants truncated in different components of the Type III‐like transport system. The resulting variants of mutated flagella architectures depending on the truncated gene are displayed in (A) and all of them lacked the flagellum filament. The microscopic observation of DnaK and FliC in these strains was performed in parallel samples to label only one protein in each sample (B). DnaK showed a distinct pattern of intracellular localisation, accumulating in spots instead of the homogeneous distribution in the membrane area observed in the PA14 wild type depicted in Figures 2A and 4B. The *fliC*::MAR2xT7 mutant, which solely lacks FliC, displayed a heterogeneous pattern of intracellular levels of DnaK protein (B). The *flgK*::MAR2xT7 mutant, which is able to assemble the hook but unable to polymerise the filament (A) displayed the highest amounts of DnaK intracellular accumulation (B). The *flgI*::MAR2xT7 mutant, affected in the flagellar basal body assembly (A), also accumulated the protein intracellularly (B). Generally, FliC was found in very low amount in the wild type strain inside the bacterial cell (Figure 2C) and was labelled only in the flagellum (Figures 2A and 4B). FliC was absent intracellularly in the *fliC*::MAR2xT7 mutant and almost not present in *flgK*::MAR2xT7 (B). However, larger amounts of FliC were found intracellularly and concentrated in spots in several *flgI*::MAR2xT7 bacterial cells (B). Bars, 2 μm.

Considering the tight association found between FliC and DnaK, the subcellular location of DnaK in several transposon mutants with inactivated genes for essential structural components of the flagellum (highlighted in red in Figure 4A) was investigated. Mutations in the filament *fliC* gene (*fliC*::MAR2xT7) itself, and in the *flgK* gene (*flgK*::MAR2xT7) encoding the junction protein between the hook and the filament, should prevent final flagellum assembly, but should not disturb the onset of the Type III‐like mediated protein secretion (Figure 4A). In addition, the *flgI*::MAR2xT7 mutant represents a peptidoglycan‐embedded P‐ring truncation and thus formation only of the MS and C‐ring is possible. Thereby, protein secretion is totally prevented, although the assembly of flagellum subcomplexes consisting of the MS and C‐rings might allow protein translocation into the periplasmic space (Figure 4A). As expected, none of these structural mutants allowed for a properly assembled filament as confirmed by immunofluorescence microscopy (Figure 4B) and the *fliC*::MAR2xT7 mutant additionally lacked intracellular FliC (Figures 4B and S8). The *flgK*::MAR2xT7 mutant displayed negligible amounts of intracellular FliC (Figures 4B and S8), which indicates either extensive FliC secretion, and/or lower expression levels, and/or rapid degradation. In these transposon mutants a higher accumulation of intracellular DnaK was also observed compared with the wild type (Figures 4B and S8). In the *fliC*::MAR2xT7 mutant, DnaK was found to be concentrated in larger spots present across the entire cytoplasm of the bacterial cell suggesting co‐dependence of FliC‐DnaK export (Figures 4B and S8). Immunogold electron microscopy studies revealed that, solely in this mutant, DnaK was distributed throughout the cytoplasm and not only at the cell periphery (Figure S9). The *flgK*::MAR2xT7 mutant depicted another fluorescence pattern of intracellular DnaK accumulation in the form of smaller spots scattered around the cell periphery (Figures 4B and S8). This correlates with the immunogold localisation studies, where DnaK was clearly distributed in the membrane area, mostly clustered as 3–4 gold particles (Figure S9). On the other hand, the *flgI*::MAR2xT7 mutant, which is heavily impaired in mature flagellum assembly, showed spot‐localised intracellular presence of DnaK and FliC in contrast to the wild type (Figures 4B and S8).

These results clearly indicate that DnaK production and localisation is intimately linked to FliC production and transport, as well as proper flagellum assembly. The unexpected extracellular DnaK location reflects trafficking of the molecular chaperone across the cytoplasmic and outer membranes and the periplasm to reach the extracellular environment. It is known that FliC translocation in bacteria is mediated through the integral Type III‐like secretion system of the flagellar export apparatus with the help of several cytoplasmic chaperones (Minamino 2014). In addition, we have previously proven that FliC is also found in the periplasm interacting with DnaK, indicating an alternative transit of the two proteins from the cytoplasmic membrane to the periplasm (Borrero‐de Acuña et al. 2015).

### 3.7 DnaK Repression Impairs FliC Export and Flagellum Assembly

In the previous section, it was shown that DnaK expression correlated with proper FliC transport and filament assembling. Thus, it remained to be ascertained whether the DnaK protein is essential in vivo for the assembly and function of the flagellum machinery. To address this point, several genetic editing approaches were attempted to completely knock out the *dnaK* gene, but without success. Deletion was attempted by homologous recombination using the *sacB* counterselection (Huang and Wilks 2017) and the I‐SceI‐dependent (Martínez‐García and de Lorenzo 2011) selection systems, the CRISPR‐Cas9‐mediated genetic editing (Pankratz et al. 2023) and CRISPRi‐mediated *dnaK* gene expression interference (Batianis et al. 2020). DnaK thus seems to be an essential function in *P. aeruginosa* PA14. Finally, *dnaK* expression was repressed using an IPTG‐inducible *dnaK* anti‐sense RNA hybridisation system (Rusmini et al. 2014) to generate a conditional knock out. The repression of *dnaK* was validated by Western blotting using anti‐DnaK antibodies (Figure S10A). As expected, the wild type strain showed stable DnaK production over time, whereas the *dnaK* conditional mutant displayed a clearly reduced DnaK production 2 h after IPTG induction. After that time point, DnaK production recovered, most likely mediated by the *P. aeruginosa* ribonuclease III (RNase III) surveillance system.

**FIGURE 5:**
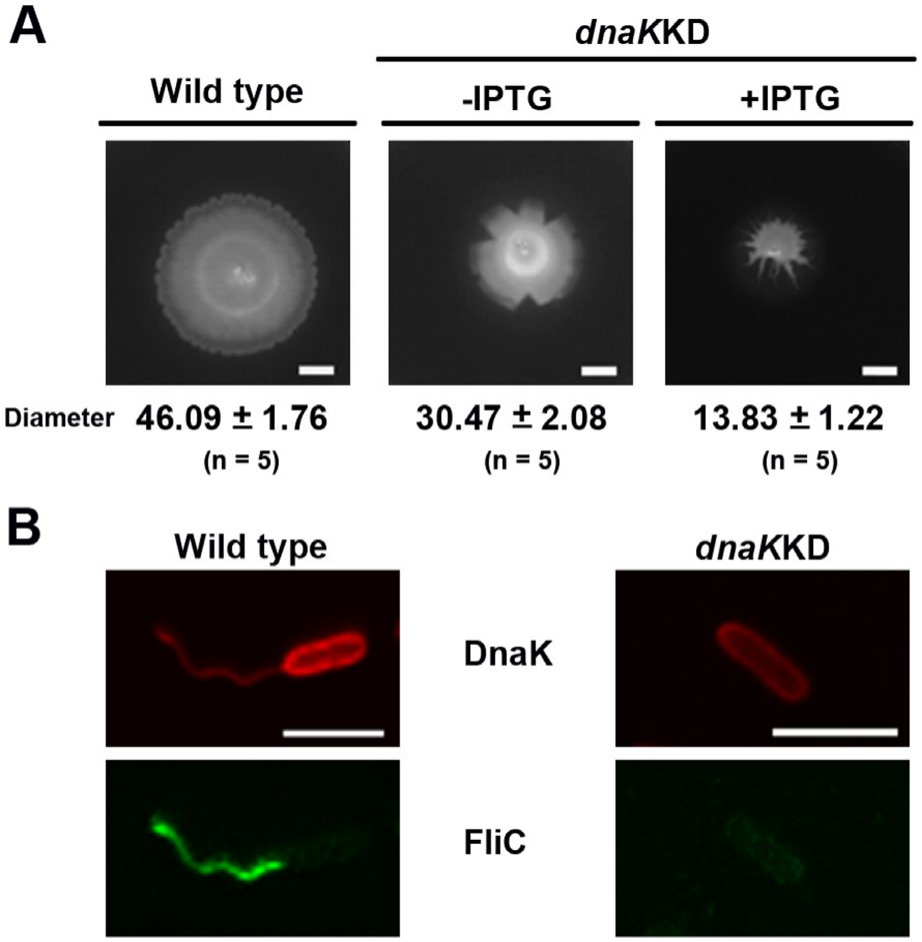
Characterisation of a *dnaK* conditional mutant (*dnaK*KD) in terms of (A) swimming motility and (B) FliC and DnaK production and localisation. Anti‐sense RNA repression of *dnaK* was activated by IPTG. In (A) the swimming motility of the wild type (left) and *dnaK* knockdown mutant (*dnaK*KD; right panels) was tested on agar swimming plates. The *dnaK* expression was repressed (+IPTG) or allowed (−IPTG) in the mutant. The diameter of the swimming halos for each strain was measured in five replicates. The *dnaK* repressed mutant displayed inefficient swimming motility. Bars, 10 mm. In (B) DnaK (red) and FliC (green) production and localisation in the wild type (left panel) and in the *dnaK*KD repressed strain when IPTG was added (right panel) was assessed by immunofluorescence as described in Figure 2A. DnaK protein level was reduced in the knockdown strain in comparison to the wild type in the bacterial cell and almost absent associated to the flagella. FliC protein was intracellularly retained to a larger extend in the *dnaK* knockdown mutant in contrast to the wild type. Bars, 10 mm in (A) and 2 μm in (B).

The swimming motility of the *dnaK* conditional mutant and the wild type were compared. For this purpose, overnight grown cultures of the aforementioned strain were point inoculated on soft agar plates used to assess motility plates with and without IPTG. As observed in Figure 5A the wild type strain presented a classical swimming halo. In contrast, the *dna*K conditional mutant displayed reduced motility activity in the plates lacking IPTG (probably due to a leaky antisense RNA promoter), and almost no motility in plates supplemented with IPTG. This indicates a major impairment of flagellar movement when *dnaK* expression is down regulated.

Immunofluorescence analyses of FliC and DnaK production and distribution across the bacterial subcellular compartments was performed 2 h after IPTG‐mediated induction of *dnaK*‐antisense RNA synthesis. As anticipated, DnaK production was visibly reduced in the conditional *dnaK* mutant in comparison to the wild type strain (Figures 5B and S10). Strikingly, a significant number of conditional *dnaK* mutant cells lacked a properly assembled flagellum, showed aberrant flagellar morphologies and, in some cases, FliC appeared to be retained intracellularly (Figure S10). Collectively, these results indicate an essential role of the DnaK protein for FliC transport and consequently flagellar filament assembly and functionality.

### 3.8 DnaK Functions in an ATP‐Independent Manner at Both Physiological and Mild Acidic pHs

The finding of DnaK secretion and association with flagella directed our investigation towards elucidation of its corresponding function. Canonically, Hsp70s, jointly with co‐chaperones such as Hsp40, assist the folding of proteins, maintain their correct conformation and prevent aggregation, via multiple events of substrate binding and release, intrinsically coupled with ATP hydrolysis cycles (Hartl, Bracher, and Hayer‐Hartl 2011). Although not yet fully understood, two major interconnected mechanisms have been recognised for DnaK. Firstly, a passive DnaK holding of the unfolded/misfolded substrate, an activity also termed ‘Holdase’, has been described. Secondly, an active unfolding of otherwise kinetically trapped unfolded/misfolded states (i.e., ‘(Un) foldase’), which in turn promotes their proper (re)folding to the native state, was reported (Slepenkov and Witt 2002; Sharma, Christen, and Goloubinoff 2009; Sharma et al. 2010; Imamoglu et al. 2020). Chaperones displaying classical holdase behaviour do not consume ATP to perform their function (Sun and MacRae 2005). However, the holdase activity of DnaK, which also involves DnaJ and GrpE, has been reported to require ATP (Slepenkov and Witt 2002).

To the best of our knowledge, there is no report thus far of significant levels of ATP in the periplasm or in the immediate vicinity of the external cell surface. Furthermore, the periplasm of Gram‐negative bacteria constitutes an acidic space (pH ~6) due to the generation of a proton gradient across the membrane (Wilks and Slonczewski 2007; Monteagudo‐Cascales et al. 2022). pH‐induced structural changes of DnaK and their effects on substrate binding were shown to be substantial only for pH‐values below 4.5 (Sehorn, Slepenkov, and Witt 2002). Despite these indications, we investigated whether DnaK might function as an ATP‐independent holdase at both neutral (pH 7) and slightly acidic environments (pH 6).

The favourable engagement of chaperones with unfolded substrates via holdase results in a shift of the clients ‘folding equilibrium’ towards the unfolded state (Zahn, Perrett, and Fersht 1996; Wood et al. 2018), as well as a slowing down of the folding reaction (Sekhar et al. 2012). To examine this holding activity, we purified recombinant DnaK protein by affinity chromatography and measured its capacity to bind an unfolded substrate and simultaneously alter its folding stability and kinetics. Initially, we intended to use FliC as substrate. However, recombinant *P. aeruginosa* FliC, while soluble, produced diverse polymeric forms under multiple test conditions, and thus was not appropriate for protein folding/unfolding experiments requiring defined stoichiometries of the tested partners. Flagellin subunit from other bacteria yielded comparable results. We therefore used for proof of principle SOD1_bar_‐G41D, a previously established Förster resonance energy transfer (FRET) folding sensor (Gnutt et al. 2019), known to bind to mammalian Hsp70 (Okado‐Matsumoto and Fridovich 2002).

**FIGURE 6:**
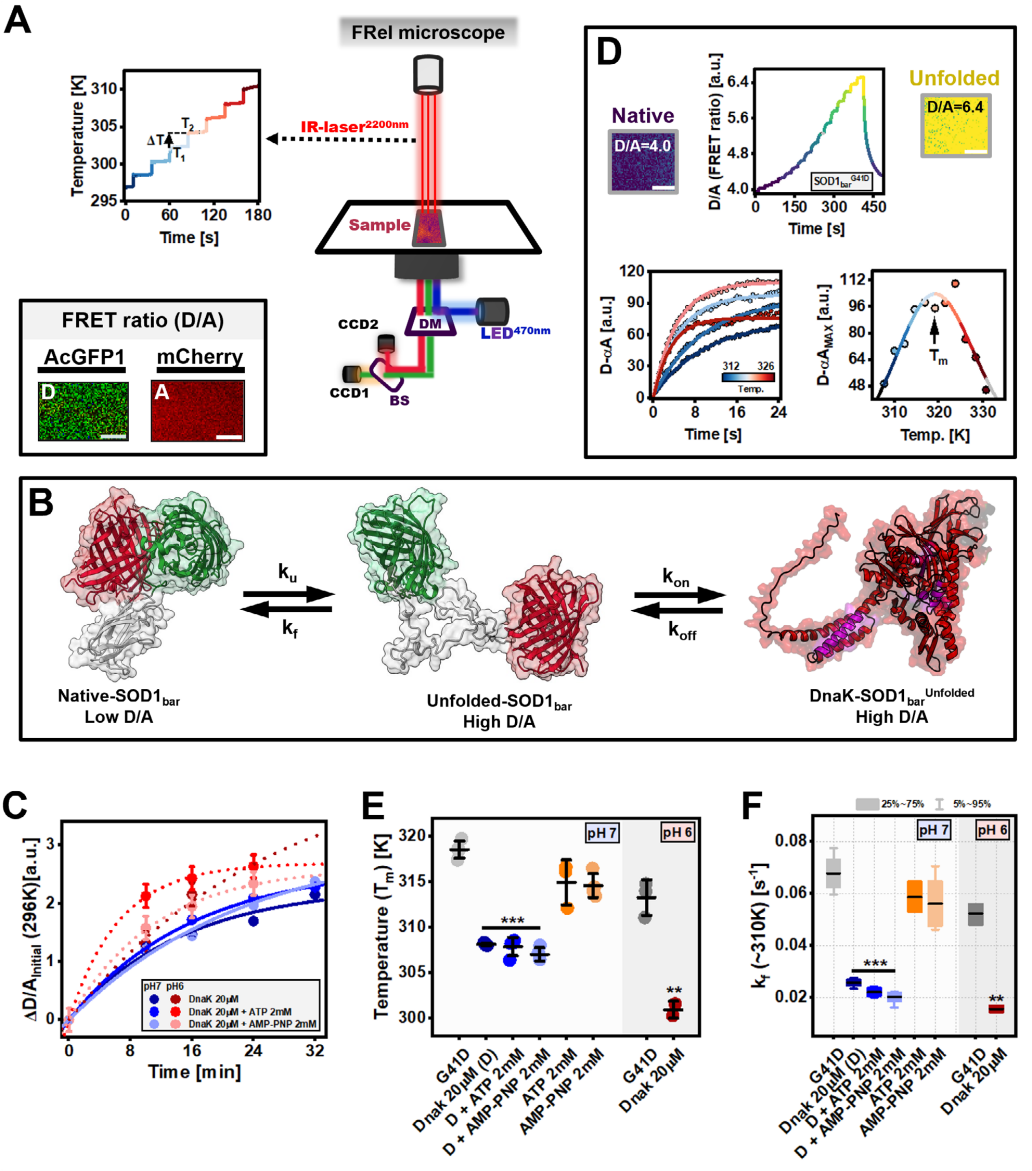
In vitro DnaK activity assay at both neutral (pH 7) and acidic (pH 6) conditions. (A) Fast relaxation Imaging (FReI) technique. D (in white) stands for donor (AcGFP1), A (in white) for acceptor (mCherry), DM for dichroic mirror and BS for bean splitter. The figure on the left shows the gradual temperature increase with time by consecutive IR‐temperature jumps (T jumps) of 25 s each and ~2.3 K (Δ*T*). (B) Cartoon representation of SOD1_bar_‐wt 3D structure with both AcGFP1 and mCherry fused to the N and C‐terminus, respectively (PDBs: 4BCZ, 3LVA and 2H5Q). At RT, SOD1_bar_ exists in equilibrium between the native and unfolded conformations, with the former being mostly populated. When DnaK (PDB: 2KHO) is added to the solution, it complexes with unfolded SOD1_bar_, thus shifting the equilibrium towards the unfolded state (*k*_f_/*k*_u_ stand for folding an unfolding rate, and *k*_on_/*k*_off_ stand for association and dissociation rate constants, respectively). Images were built using UCSF ChimeraX (Meng et al. 2023). (C) Initial FRET signal at RT (Δ*D*/*A*_Initial_ = *D*/*A*_*i* DnaK/Nucleotides_ *D*/*A*_*i* SOD1_) of SOD1_bar_‐G41D in the presence of DnaK 20 μΜ and/or 2 mM ATP, 2 mM AMP‐PNP at both pH 7 and 6. Solid lines represent the finest fit for Equation (2) (See Table S4 for details). (D) Exemplary FRET‐thermal unfolding curve of SOD1_bar_‐G41D at pH 7 (upper plot), with corresponding unfolding kinetic amplitudes (*D*‐*αA*) at different temperatures (lower left plot). Lower right plot shows fitted steady‐state unfolding amplitudes as a function of temperature, with the maximum of the curve defining *T*_*m*_. *T*_*m*_ (E) and *k*_f_ (310 K) (F) values for each tested condition with DnaK and/or ATP, AMP‐PNP. Bars represent mean values and errors are shown as standard deviations. Statistical significance tests were carried out in GraphPad Prism Version 8.0.1. One‐way ANOVA analysis was performed followed by post hoc Tukey test (** *p* ≤ 0.01, ***p ≤ 0.001). Statistical significances shown refer to SOD1_bar_‐G41D condition.

SOD1_bar_‐G41D was fused with AcGFP1 (Donor‐D) and mCherry (Acceptor‐A) fluorescent proteins at the N‐ and C‐termini, respectively (Figure 6A,B). At room temperature (RT, 296.2 K) and pH 7, the modified standard state free energy of folding (Δ*G*_*f*_^0’^) for SOD1_bar_‐G41D is −8.7 ± 1.0 kJ mol^−1^, indicating that the protein existed mostly in its native conformation (folded fraction of ~97%) (Gnutt et al. 2019). However, in the presence of DnaK, transition of SOD1_bar_‐G41D to the unfolded state was promoted, as indicated by an increase in the FRET ratio (*D*/*A*) (Figure 6A,B). First, we monitored the change in FRET intensity (Δ*D*/*A*_Initial_ = *D*/*A*_*i* DnaK_ *D*‐*A*_*i* SOD1_) when DnaK (20 μM) was combined with SOD1_bar_‐G41D (10 μM) at RT, pH 7 over a 32 min period (Figure 6B). The plot revealed a significant increase of the Δ*D*/*A*_Initial,_ reaching a plateau after ~15 min (Figure 6C and Table S4). Remarkably, addition of ATP or its non‐hydrolysable analogue Adenylyl‐imidodiphosphate (AMP‐PNP) in excess did not cause a significant change on *k*_obs_ or Δ*D*/*A*_Initial_^MAX^ values (Figures 6C and S11A, Table S4), suggesting that the nucleotides have a negligible effect on the binding reaction. We also confirmed that the addition of ATP or AMP‐PNP alone did not cause any time‐dependent change of SOD1_bar_‐G41D initial D/A (Figure S11B). Together, these results suggest that DnaK binds to unfolded SOD1_bar_‐G41D in an ATP independent manner.

To further confirm DnaK‐binding and consequent destabilisation of SOD1_bar_‐G41D, we measured the change in melting temperature (*T*_*m*_) of the protein caused by DnaK (20 μM) in the presence or absence of these nucleotides (2 mM) at pH 7, using the fast relaxation imaging (FReI) technique (Figure 6A) (Ebbinghaus et al. 2010). FReI measures the time of evolution of the FRET‐pair signal, along with a gradual heating induced by fast (millisecond) IR‐temperature jumps (T‐jumps), in set intervals of 25 s (Figure 6A). Specifically, SOD1_bar_‐G41D was heated incrementally by total of 16 Tjumps (~2.3 K each), covering a range of temperatures from 296.2 to 333 K. Refolding of the protein was promoted by cooling it down at room temperature during 60 s after each unfolding experiment. Figures 6D and S11C show exemplary normalised thermal unfolding FRET ratio curves as a function of time for SOD1_bar_‐G41D alone or in combination with DnaK. Steady‐state unfolding kinetic amplitudes (D‐αA_max_) for each Tjump were then plotted versus temperature and fitted to a two‐state folding model (Equation 3) to determine *T*_*m*_ (Figure 6D). As expected for the DnaK holdase activity, we found a significant destabilisation (*T*_*m*_ decreased by ~10 K) of SOD1_bar_‐G41D when DnaK was present (Figure 6E). Addition of ATP or AMP‐PNP did not affect the overall extent of the destabilisation mediated by the chaperone alone (Figure 6E). SOD1_bar_‐G41D folding rates (*k*_*f*_) at 310 K (~37°C) were also significantly reduced (~3 fold) in the presence of DnaK (Figure 6F), which supports an ATP‐independent complex formation between the chaperone and unfolded protein.

After characterising the ATP‐independent holdase function of DnaK at intracellular pH, we performed the equivalent experiment at pH 6, a condition resembling that of the periplasmic space. We recorded SOD1_bar_‐G41D Δ*D*/*A*_Initial_ curves after DnaK (20 μM, (1:2)) incubation with and without ATP/AMP‐PNP (2 mM) (Figure 6C). At this pH, DnaK stimulated a significant increase in the initial FRET ratio, reaching a maximum after ~27 min (Figures 6C and S11A, Table S4). Also, a substantial destabilisation of SOD1_bar_‐G41D by DnaK (Figures 6E and S11C) was observed, accompanied by slower folding rates (Figure 6F). Overall, these findings reinforce the hypothesis of DnaK association with unfolded SOD1_bar_‐G41D at pH 6 in the absence of ATP. In fact, when ATP or AMP‐PNP were added to DnaK+SOD1_bar_‐G41D samples, k_obs_ increased four and twofold, while Δ*D*/*A*_Initial_^MAX^ decreased by ~1.7‐fold, respectively (Figures 6C and S11A, Table S4), an indication that both nucleotides affect the complex formation between the proteins at this pH. Moreover, in the presence of DnaK + ATP/AMP‐PNP, quantification of folding stability (*T*_*m*_) and kinetics (*k*_*f*_) was not possible, given the linear unfolding kinetics traces exhibited by the *D*/*A* versus time thermal unfolding curves (Figure S11D). In addition, changes in binding affinities of DnaK:substrate in the apo or nucleotide‐bound states at these more acidic pHs cannot be excluded. Therefore, further studies are required to quantitatively estimate the effect of the ATP cycle on DnaK:SOD1_bar_‐G41D‐binding reaction at pH 6. In conclusion, our results show that DnaK can function as a classical holdase under both neutral pH and also mild acidic conditions.

## 4 Discussion

The work reported here suggests that the DnaK chaperone is an essential interaction partner of FliC, the major subunit of the flagellum filament of *P. aeruginosa* and that these two proteins interact in multiple ways in different contexts, which dictate the conformational requirements of FliC for its traversal of the inner and outer membranes and periplasm, and its assembly into flagellar filaments. Unexpectedly, DnaK was found to be incorporated in the mature extracellular flagellum.

Our previous work revealed that the DnaK–FliC partnership is crucial for flagellar movement under nitrate‐respiring anaerobic conditions (Borrero‐de Acuña et al. 2015). In the current work, the highly abundant DnaK–FliC complex was readily isolated from bacterial cells grown under aerobic conditions by immunoprecipitation (Table 1). SPOT membrane peptide arrays and computational modelling suggested that the holdase domain of DnaK interacts with the C‐terminal D0 domain of FliC. Given that the FliC protein, as the major component of the self‐assembling filament polymer of the flagellum, has the intrinsic ability to self‐polymerise, it is likely that this process must be prevented prior to assembly of the mature flagellum. In fact, several attempts to hyperproduce recombinant FliC in different bacteria resulted in the formation of inclusion bodies (Khani et al. 2019). Holdase‐mediated DnaK interaction with FliC preventing FliC self‐polymerisation probably occurs immediately after ribosomal synthesis. As we observed DnaK in intact cells located close to the inner surface of the inner membrane, it seems likely that ribosomal FliC production and immediate DnaK binding take place at this location (Figures 2 and S9).

In *Salmonella*, the flagellum‐dedicated chaperone FliS binds to the C‐terminal disordered region (D0) of FliC; in the absence of FliS, an aberrantly short filament forms (Ozin et al. 2003; Muskotál et al. 2010). FliC attached to FliS binds to the transmembrane gate protein FlhA; the structure of the FlhA–FliS–FliC complex has been characterised in *Salmonella* and shown to mediate FliC export through the flagellum (Xing et al. 2018). The *P. aeruginosa* FliS (PA14_50250) protein was not found as a constituent of the DnaK‐FliC complex studied in this work, which may suggest that DnaK and FliS cannot simultaneously bind to FliC. Considering that *Salmonella* has several peritrichous flagella and *P. aeruginosa* has a single flagellum, the FliC–DnaK interactions might be specific to *Pseudomonas*, while the FliC–FliS interplay might be distinctive of *Salmonella*. Further functional correlation between DnaK and FliC have been observed in other Gram positive and Gram‐negative bacteria, including pathogens. In *Clostridioides difficile* disruption of *dnaK* results in impaired motility due to a FliC‐deficient phenotype. An insertional mutant in the *dnaK* gene was found to be non‐motile producing fourfold lower expression of the *fliC* gene and lacking flagella on the cell surface (Jain et al. 2017). In *E. coli* the inactivation of *dnaK* also results in a non‐motile phenotype (Shi et al. 1992). However, a detailed study of the DnaK–FliC molecular interplay such that observed in *P. aeruginosa* and described here was not conducted in other species.

The observed flexibility of DnaK–FliC interaction allows the nucleotide binding domain to rotate upon itself and interact with new stretches of the D1 and D0 domains of FliC. Assuming that DnaK also reaches the extracellular environment via the Type III‐like export system, all of these conformational changes may be necessary for FliC‐DnaK unfolding prior to their insertion and passage through the 2‐nm export channel inside the flagellum. How the coordinated unfolding and transport of both proteins occurs, and at which stoichiometry they are exported in *Pseudomonas*, remains to be determined. Compared to the several thousand FliC molecules required for flagellum formation only a few DnaK molecules were found attached to the flagellum surface in an almost regularly pattern (Figure 2). In addition, a swinging‐like movement of the DnaK molecule was found induced during the interactions between its nucleotide binding domain (ATPase) with the stretches of FliC exposed to the outside in an assembled filament (domains D2 and D3). A sterically favoured intercalation arrangement of DnaK into a fully assembled reconstructed filament was confirmed by computational analyses (Figure 3B). This provides evidence of another type of FliC‐DnaK interaction involved in DnaK attachment to the flagellum.

Our studies indicate that the DnaK chaperone is present in different bacterial compartments: the cytoplasm, the periplasmic space and the extracellular milieu. To tackle the possible routes that DnaK and FliC undertake to reach the periplasm and beyond, several transposon mutants were investigated. DnaK and FliC locations were analysed in mutants deficient in the general SecYEG and twin arginine (Tat) periplasm translocation systems (Minamino 2014), and mutants in Xcp‐T2SS proteins, all of which mediate protein secretion across the outer membrane (Costa et al. 2015). Although DnaK and FliC abundances were slightly increased in the cytoplasm of the mutants tested, both proteins were always detected extracellularly associated with the flagellum, as determined by immunofluorescence microscopy (unpublished observations). These results suggested that DnaK might use alternative translocation routes.

Recently, with the implementation of electron cryo‐tomography (ECT) (Oikonomou and Jensen 2017), imaging evidence was provide showing the in situ structures and differences of various bacterial flagellar motors within intact cells. ECT studies have revealed that, while the flagellar motor core structure is conserved, it has evolved in many species resulting in specific adaptations to different environmental conditions (Kaplan, Ghosal, et al. 2019; Kaplan, Subramanian, et al. 2019). In these studies, it was demonstrated that species that harbour a single and polar flagellum possess elaborated P‐ and L‐rings (Figure 4A). Particularly, and only, *L‐ pneumophilia* and *P. aeruginosa* have an extra periplasmic ring surrounding the P‐ring (Kaplan, Ghosal, et al. 2019; Kaplan, Subramanian, et al. 2019). In addition to fully assembled flagella, these species showed stable outer‐membrane complexes, named PL subcomplexes, because similar to the P‐ and L‐rings (Figure 4A) (Kaplan, Ghosal, et al. 2019; Kaplan, Subramanian, et al. 2019). These complexes may have a specific function or, considering that are present in the periplasm, they may be difficult to degrade. In addition, independent inner‐membrane sub‐complexes with an intact MS‐ring were also found (Kaplan et al. 2022a, 2022b). A novel hat‐shaped structure was detected embedded in the inner membrane of bacteria, including *P. aeruginosa* , harbouring the flagellar Type III secretion system (Kaplan et al. 2022a, 2022b). In conclusion, ECT imaging provide clear evidence of the complexity and plasticity of the flagellar apparatus that might contribute to the protein translocation to and out from the periplasm and might explain the presence of both DnaK and FliC in the periplasm and extracellular environment.

DnaK, with its partners DnaJ and GrpE, using ATP as a source of energy, is generally understood as a protein involved in the proper folding of nascent proteins or misfolded proteins. However, observations reported from a variety of organisms suggest that the DnaK polypeptide, its molecular chaperone function put aside, is also interacting with a variety of proteins with widely different functions. Analysis of the functional cycle of the DnaK–DnaJ complex shows that the first step of the cycle involves DnaK alone, allowing the protein to interact with other proteins, in the absence of ATP (Figure S12).

In addition to the DnaK transport question, an environment deprived of ATP such as that of the periplasm and the extracellular milieu poses a challenge for DnaK to conduct its canonical ATP‐dependent chaperone foldase activity. But, is the foldase activity outside of the cell required, or is the pertinent DnaK function that of an ATP‐independent holdase? We have demonstrated DnaK holdase activity under extracellular conditions in the absence of ATP. DnaK binds ATP very tightly (*K*_*d*_ = 1 nM) (Russell, Jordan, and McMacken 1998), and any extracellular ATP binding to DnaK would likely interfere with the swinging‐like motion enabled by transient interactions between the NBD of DnaK and exposed FliC domains in the flagellum. Thus, the extracellular ATP‐independent DnaK function might be fundamental for the correct assembly of the flagellum. It is conceivable that DnaK at the flagellum tip aids proper folding of FliC and reduces incorrect assembly caused by inappropriate folding states of FliC. Secondly, DnaK might serve as a structural element of the flagellum, perhaps contributing to its elasticity or flexibility, and hence to overall flagellum function. Thirdly, DnaK might contribute to filament assembly and disassembly dynamics by, for example, providing protease attachment points for flagellum depolymerisation (an intracellular cooperativity of DnaK and proteases like ClpB has been reported (Mazal et al. 2019).

More broadly, it should be emphasised that protein interaction surfaces offer a wealth of opportunities for design and development of small molecule agonists and antagonists for the function involved, for both investigative and applied purposes (Timmis 2018). Given that motility of *P. aeruginosa* , and hence FliC, is essential for virulence (Garcia et al. 2018), as is DnaK as we have demonstrated, their interaction surfaces would seem to offer precise targets for the design of new drugs targeting bacterial virulence.

## 5 Conclusions

The DnaK protein (heat shock protein HSP70) is the central cellular component for folding and unfolding of cytoplasmic proteins. In this work, we demonstrate and explain for the first time the essential extracellular function of DnaK during the formation of the bacterial swimming apparatus, the flagellum in the pathogenic bacterium *Pseudomonas aeruginosa* . Disrupting the dynamic interactions occurring between DnaK and FliC leads to the retention of the flagellin within the cell unable to be exported through the Type III‐like secretion apparatus, flagellum assembly is impaired, and the bacterium fails to swim. Unprecedently, our studies reveal an ATP‐independent DnaK chaperoning function resembling extracellular conditions wherein this chaperone is secreted and associated to the flagellum filament. This study encourages to pursue further insights into structural and molecular function of the DnaK as a component of the flagellum machinery. Collectively, our results add to the well‐established knowledge about intracellular DnaK function, a novel extracellular activity which might serve as future drug target.

## Author Contributions

**Gabriella Molinari:** conceptualization, data curation, formal analysis, investigation, project administration, supervision, validation, visualization, writing – original draft, writing – review and editing. **Sara S. Ribeiro:** data curation, investigation, methodology, validation, visualization, writing – original draft. **Katrin Müller:** investigation. **Benjamin E. Mayer:** data curation, formal analysis, investigation, methodology, software, validation, visualization, writing – original draft. **Manfred Rohde:** investigation, resources, visualization. **Alejandro Arce‐Rodriguez:** investigation. **Juan José Vargas‐Guerrero:** investigation. **Albert Avetisyan:** investigation, methodology. **Josef Wissing:** data curation, investigation. **Werner Tegge:** data curation, investigation. **Lothar Jänsch:** data curation, resources. **Mark Brönstrup:** resources. **Antoine Danchin:** conceptualization, writing – review and editing. **Martina Jahn:** resources. **Kenneth N. Timmis:** conceptualization, writing – review and editing. **Simon Ebbinghaus:** funding acquisition, methodology, resources, software, supervision, writing – original draft. **Dieter Jahn:** conceptualization, formal analysis, funding acquisition, project administration, resources, supervision, writing – original draft, writing – review and editing. **José Manuel Borrero‐de Acuña:** conceptualization, data curation, formal analysis, funding acquisition, project administration, resources, supervision, validation, visualization, writing – original draft, writing – review and editing.

## Conflicts of Interest

The authors declare no conflicts of interest.

## Supporting information


Video S1

## Data Availability

This study includes no data deposited in external repositories. Data are available on request from the authors.
