# Correction to ‘Multiple Chaperone DnaK–FliC Flagellin Interactions Are Required for *Pseudomonas aeruginosa* Flagellum Assembly and Indicate a New Function for DnaK’

**DOI:** 10.1111/1751-7915.70138

**Published:** 2025-03-26

Molinari, G., S. S. Ribeiro, K. Müller, et al. 2025. “Multiple Chaperone DnaK–FliC Flagellin Interactions Are Required for *Pseudomonas aeruginosa* Flagellum Assembly and Indicate a New Function for DnaK.” Microbial Biotechnology 18, no. 2: e70096. https://doi.org/10.1111/1751‐7915.70096.

In paragraph 4 of the ‘3.8 | DnaK Functions in an ATP‐Independent Manner at Both Physiological and Mild Acidic pHs’ subsection in the ‘Results’ section, the equation ‘Δ*D*/*A*_Initial_ = *D*/*A*_iDnaK_ *D − A*_iSOD1_’ was incorrect. This should have read: ‘*ΔD/A*_Initial_ *= D/A*_iDnaK_ *− D/A*_iSOD1_’.

In caption of Figure 6 (C) of the ‘Results’ section, the equation ‘Δ*D*/*A*_Initial_ = *D*/*A*_iDnaK/Nucleotides_ *D*/*A*_iSOD1_’ was incorrect. This should have read: ‘Δ*D*/*A*_Initial_ = *D*/*A*_iDnaK/Nucleotides_ − *D*/*A*_iSOD1_’.

We apologise for this error.

